# Blockade of EP3 attenuates cardiac fibrosis by suppressing TGF-β1-Smad2/3 signaling through the Smurf2-mediated Smad7 ubiquitination

**DOI:** 10.1016/j.mmr.2026.100071

**Published:** 2026-10-06

**Authors:** Lan Ye, Hu Xu, Hui Tang, Qing-Wei Li, Xiu-Hui Mao, Er-Jiao Qiang, Jia-Yao Zhang, Bo Liang, Li-Hong Chen, You-Fei Guan, Xiao-Yan Zhang

**Affiliations:** a Advanced Institute for Medical Sciences, Dalian Medical University, Dalian 116044, Liaoning, China; b Health Science Center, East China Normal University, Shanghai 200241, China; c Department of Pathology, Shanghai General Hospital, Shanghai Jiaotong University School of Medicine, Shanghai 200080, China

**Keywords:** E-type prostanoid receptor 3 (EP3), Cardiac fibrosis, TGF-β signaling, Smad7, Ubiquitination

## Abstract

**Background:**

Sustained β-adrenergic activation induces cardiac fibrosis, which is characterized by excessive deposition of extracellular matrix (ECM) secreted by activated cardiac fibroblasts (CFs). Prostaglandin E_2_ (PGE_2_) is a dominant cyclooxygenase metabolite of arachidonic acid and exerts important biological actions by activating four G-protein-coupled receptors, including E-type prostanoid receptors 1–4 (EP1–EP4). The present study aimed to investigate the role of the EP3 receptor in isoproterenol (ISO)-induced cardiac fibrosis.

**Methods:**

To elucidate the role of EP3 in cardiac fibrosis, the global *EP3* gene knockout mice (EP3^−/−^) and the LoxP-Cre system-based CF-specific *EP3* gene knockout mice (EP3^cKO^) or cardiomyocyte (CM)-specific *EP3* gene knockout mice (EP3^mKO^) were subcutaneously injected with ISO [5 mg/(kg·d)] for 7 d to establish a cardiac fibrosis model. In parallel, the EP3 agonist sulprostone and the EP3 antagonists DG041 and L798106 were used to elucidate the underlying mechanisms. Finally, the therapeutic potential of the EP3 antagonist DG041 was evaluated in the context of cardiac fibrosis.

**Results:**

Echocardiography assay showed that both EP3^−/−^ and EP3^cKO^ mice exhibited a significant improvement in cardiac diastolic function. Histological staining revealed that EP3^−/−^ and EP3^cKO^, but not EP3^mKO^, exhibited a significant attenuation of ISO-induced CF activation and ECM accumulation. Further investigation uncovered that EP3 was highly expressed in the CFs rather than CMs. Inhibition of EP3 in the CFs suppressed transforming growth factor-β1 (TGF-β1)-triggered phosphorylation of small mother against decapentaplegic (Smad)2/3 and the production of ECM proteins. Mechanistically, the blockade of EP3 in the CFs activated the phosphatidylinositol 3-kinase (PI3K)/protein kinase B (Akt) pathway, which promoted the degradation of Smad ubiquitination regulatory factor 2 (Smurf2) and subsequently suppressed the ubiquitination of Smad7, an inhibitory Smad, leading to an increase in the protein level of Smad7. Furthermore, the administration of an EP3 antagonist, DG041, markedly improved ISO-induced cardiac diastolic dysfunction and fibrosis.

**Conclusions:**

This study demonstrates that CF-EP3 contributes to β-adrenergic activation-induced cardiac fibrosis and target of EP3 represents a potential therapeutic strategy for the treatment of cardiac fibrosis.

## 1 Background

Cardiac fibrosis is a response to a variety of cardiac insults and is characterized by an excessive deposition of extracellular matrix (ECM) proteins, particularly collagens, within the myocardial tissue, leading to stiffening and impaired cardiac function [1], [2]. Sustained β‑adrenergic receptor (β‑AR) activation, unlike its cardiotonic role under physiological conditions, induces cardiac pathophysiological remodeling, including hypertrophy and fibrosis, through paracrine factors [e.g., interleukins, connective tissue growth factor (CTGF), vascular endothelial growth factor (VEGF), and transforming growth factor‑β (TGF‑β)] released from cardiomyocytes (CMs), which in turn regulate the function of cardiac fibroblasts (CFs) [3], [4], [5], [6], [7]. Upon stimulation, the CFs are activated and differentiated into myofibroblasts, which exhibit secretory, matrix-producing, and contractile characteristics and serve as the primary source of structural ECM proteins in fibrotic hearts [8], [9]. However, although treatment with β-blockers has improved many clinical outcomes, the adverse effects are also non-negligible because of the diverse signaling transduction of β-AR [10], [11]. To date, specific anti-fibrotic therapies are not available in the clinic [12]. Therefore, it is essential to uncover the underlying mechanisms of cardiac fibrosis induced by sustained β-adrenergic activation and to identify potential therapeutic targets.

TGF-β is a multifunctional cytokine that plays a critical role in regulating cellular growth, differentiation, and immune responses [13]. The TGF-β pathway is a pivotal regulator of cardiac fibrosis, promoting the differentiation of the CFs into myofibroblasts and enhancing collagen synthesis [7], [14]. The TGF-β signaling pathway is initiated when TGF-β ligands bind to type II TGF-β receptors, leading to the recruitment and phosphorylation of type I receptors. This activates intracellular phosphorylation of small mother against decapentaplegic (Smad)2 and Smad3, which promotes their translocation to the nucleus, where they regulate their target gene expression [15]. To counteract persistent signaling, the pathway is subjected to negative feedback regulation primarily mediated by the inhibitory Smads, such as Smad6 and Smad7, which are induced upon pathway activation and function to terminate signal transduction [16]. Importantly, the turnover of Smad7 is tightly controlled by post‑translational modifications. Smad ubiquitination regulatory factor 2 (Smurf2), as an E3 ubiquitin ligase of Smad7, induces Smad7 ubiquitination and proteasomal degradation [17]. Through this dynamic interplay between Smad2/3‑driven pro‑fibrotic signaling and Smad7‑mediated feedback inhibition, the TGF‑β pathway maintains a finely tuned balance, the disruption of which may contribute to pathological fibrotic remodeling. Thus, understanding the molecular circuitry governing Smad7 stability and its antagonism of Smad2/3 activity is critical for deciphering the regulatory logic of TGF‑β signaling in cardiac fibrosis.

Prostaglandin E_2_ (PGE_2_) is a bioactive lipid mediator involved in various physiological and pathological processes, including inflammation, vascular regulation, and platelet aggregation [18]. PGE_2_ exerts its effects through 4 distinct G-protein-coupled receptors: EP1, EP2, EP3, and EP4. The EP3 receptor is unique in its ability to couple to multiple G-proteins, leading to diverse downstream effects [19]. Its activation is primarily associated with vasoconstriction, platelet aggregation, and inhibition of cyclic adenosine monophosphate (cAMP) production [19], [20]. These actions have made the EP3 receptor a focal point of research in conditions such as atherosclerosis, myocardial infarction, and thrombosis [19], [21]. It has been reported that PGE_2_ through its EP3 receptor reduces contractility in isolated CMs and in the whole heart [22]. Additionally, CM-specific overexpression of the EP3 receptor leads to decreased cardiac function in a mouse model of myocardial infarction [23]. Animal models have demonstrated that overexpression of the EP3 receptor activates calcineurin and promotes hypertrophy of the murine heart [24]. Targeted gene deletion or pharmacological inhibition of EP3 has been shown to protect against cardiac remodeling and improve post-myocardial infarction in models of cardiac injury [25]. Given these findings, the EP3 receptor has emerged as a potential therapeutic target for cardiovascular diseases. However, the role of the CF-EP3 has not been clarified.

In this study, we aimed to elucidate the role of the CF-EP3 in sustained β‑adrenergic activation‑induced cardiac fibrosis and to characterize the underlying mechanisms. To this end, we employed both pharmacological and genetic approaches in a mouse model of isoproterenol (ISO)‑induced cardiac fibrosis. Furthermore, we sought to evaluate the therapeutic potential of the selective EP3 antagonist DG041 in mitigating cardiac fibrotic remodeling.

## 2 Methods

### 2.1 Animals

A total of 127 male C57BL/6 J mice (10-to 12-week-old) were used in the experiments, of which 30 wild-type (WT) mice and 98 neonatal Sprague-Dawley (SD) rats (1–3-day-old) were purchased from the multifunctional platform for innovation of East China Normal University; the others were obtained through breeding [26]. The EP3-Loxp mice (EP3^f/f^) were constructed by Cyagen Biotechnology Co., Ltd. (China). The EP3^f/f^ mice were crossed with the S100 calcium binding protein A4 (S100A4)^Cre+^ mice (JAX stock #030644) to obtain CF-specific *EP3* gene knockout mice (EP3^cKO^). CM-specific *EP3* gene knockout mice (EP3^mKO^) were obtained by crossing the EP3^f/f^ mice with the α-Myosin heavy chain (α-MyHC)^Cre+^ mice (JAX stock #011038). The genotyping primers are listed in **Additional file 1:** Table S1. All animal care and experimental procedures were reviewed and approved by the Animal Care and Use Review Committee of East China Normal University (m+R20241108). Mice were maintained in an environment with controlled temperature (22±1) ℃ and relative humidity (50±5)% on a 12-hour light/dark cycle and with free access to sterile food and water. The study conformed to the Guide for the Care and Use of Laboratory Animals published by the US National Institutes of Health.

### 2.2 ISO-induced cardiac fibrosis model and drug treatment

Subcutaneous injection of β-AR agonist, ISO [5 mg/(kg·d); 420355, Sigma-Aldrich, USA] for 7 d was performed to establish a model of cardiac fibrosis, in accordance with previously described methods [27]. Saline (5 ml/kg)-injected mice served as control groups. For the pharmacological experiment, male wild-type (WT) mice were administered the EP3 antagonist DG041 [20 mg/(kg·d); T15108, Target Mol, USA] by subcutaneous injection one day before ISO injection and throughout the ISO treatment. DG041 was dissolved in a vehicle consisting of DMSO:PEG400:saline (1:6:3, v/v/v) to a final concentration of 5 mg/ml. At the end of the treatment, body weight was measured, and then echocardiography was performed to determine cardiac function. Finally, all mice were euthanized with an overdose of CO_2,_ and the hearts were collected for further analyses.

### 2.3 Isolation and culture of primary neonatal rat cardiomyocytes and cardiac fibroblasts

CMs and CFs were isolated and cultured from 1–3-day-old neonatal SD rat pups as described previously [28]. In brief, the hearts were removed and cut into a size of 1 mm^3^ and digested with trypsin and collagenase I, then CMs were obtained and maintained at 37 ℃ in humidified air containing 5% CO_2_. Dissociated cells were plated on 100-mm culture dishes in DMEM supplemented with 10% fetal bovine serum plus 1% penicillin and streptomycin for 2 h. The attached cells, termed CFs, were passaged once or twice before further use. The unattached CMs were plated on fresh plastic dishes, and 100 μmol/L bromodeoxyuridine was included to prevent fibroblast proliferation. CMs were directly used for experiments. Both CM and CF cultures were switched to serum-free DMEM for 12 h before treatment.

### 2.4 Echocardiography

Mice were mildly anesthetized with 1%–2% isoflurane. Cardiac diastolic function was evaluated using a high-resolution echocardiography system (Vevo 3100, Visual Sonics, Inc., Canada) at the apical four-chamber view under Doppler color mode. For the assessment of cardiac systolic function, parasternal short-axis views were obtained using B-mode imaging. Subsequently, an M-mode cursor was aligned perpendicularly to the left ventricular walls at the level of the maximum dimension in both end-diastole and end-systole, and M-mode tracings were recorded. The data were analyzed using Vevo 3100 software (version 1.5.0). Temperature and heart rate of mice were monitored and stabilized at ranges of (37.0±0.5) ℃ and (500±30) beats/min, respectively.

### 2.5 Histopathology and immunohistochemistry analyses

The heart ventricular tissues were fixed in 4% paraformaldehyde (PFA) for 24 h (4 ℃), then embedded in paraffin and cut into 4 μm sections. Hematoxylin-eosin (H&E) staining was performed for histopathology. Masson trichrome staining and Sirius Red staining were used to evaluate fibrosis of the heart. H&E staining, Masson trichrome staining, and Sirius Red staining were performed according to standard protocols. For immunohistochemical staining, cardiac sections were performed using a horseradish peroxidase-3’-3’-diaminobenzidine. Primary antibodies against α-smooth muscle actin (α-SMA; 1:1000; A5228, Sigma-Aldrich, USA) or collagen III (1:200; 22734-1-AP, Proteintech, USA) were used. Then, the sections were counterstained with hematoxylin and sealed with neutral resin. To quantify CM area, immunohistochemically positive area, and the percentage of fibrotic area, the heart section images were quantified by ImageJ.

### 2.6 Immunofluorescence staining

The ventricular sections were deparaffinized in xylene, rehydrated, and subjected to antigen retrieval and treated with a tissue spontaneous fluorescence quencher (G1221, Servicebio, China). Primary neonatal rat CFs (detailed culture methods are provided in “isolation and culture of primary neonatal rat cardiomyocytes and cardiac fibroblasts” section) and sections were permeabilized in 0.1% Triton X-100, incubated with 5% bovine serum albumin (BSA) in phosphate buffer saline (PBS) for 60 min to block non-specific antibody binding, then incubated overnight at 4 ℃ with primary antibodies against collagen III (1:200; 22734-1-AP, Proteintech, USA), cardiac troponin I (cTnT; 1:1000; 68300-1-lg, Proteintech, USA), α-SMA (1:1000; A5228Sigma-Aldrich, USA) or Smad3 (1:200; Cell Signal Technology, USA). After incubation with Alexa Fluor 488- or Alexa Fluor 594-conjugated secondary antibodies (ZSGB-BIO, Beijing, China) diluted 1:200 for 1 h at room temperature in the dark, the slides were counterstained with 4,6-diamidino-2-phenylindole (DAPI) for 5 min to visualize the nuclei. The images were captured with a microscope (DM4B, Leica, Germany) or a confocal microscope (SP8, Leica, Germany).

### 2.7 Western blotting

Ventricular heart tissue and cell lysates were subjected to sodium dodecyl sulphate-polyacrylamide gel electrophoresis (SDS-PAGE) and blotted on nitrocellulose membranes. The membranes were then incubated with antibodies against collagen III (22734-1-AP, Proteintech, USA) with 1:1000 dilution, α-SMA (A5228, Sigma-Aldrich, USA) with 1:5000 dilution, EP3 (101760, Cayman, USA) with 1:500 dilution, eukaryotic translation initiation factor 5 (eIF5; Proteintech, 11155-1-AP, USA) with 1:1000 dilution, p-Smad2 (18338S, Cell Signal Technology, USA) with 1:1000 dilution, p-Smad3 (9520S, Cell Signal Technology, USA) with 1:1000 dilution, Smad2 (5339S, Cell Signal Technology, USA) with 1:1000 dilution, Smad3 (9523S, Cell Signal Technology, USA) with 1:1000 dilution, Smad7 (ab272928, Abcam, UK) with 1:1000 dilution, Smurf2 (18038-1-AP, Proteintech, USA) with 1:1000 dilution, and ubiquitin (sc-8017, Santa, USA) with 1:500 dilution. The membranes were incubated with either anti-rabbit (AS014, ABclonol, China) or anti-mouse (AS003, ABclonol, China) HRP-conjugated secondary antibodies for 1 h. Finally, the membranes were incubated with SuperLumia ECL Plus HRP Substrate Reagent (SQ201, Epizyme, China), and the signals from immunoreactive bands were visualized using a Chemiluminescent Imaging System (Tanon 5200, Tanon Science & Technology, China). Densitometric quantification of protein bands was performed using ImageJ.

EP3 antagonists DG041 (T15108, Target Mol, USA) and L798106 (244101-02-08, Cayman Chemical, USA), EP3 agonist sulprostone (60325-46-4, Cayman Chemical, USA), and TGF-β receptor kinase inhibitor SB-431542 (HY-10431, Med Chem Express, USA) were all dissolved in DMSO and used for all cell experiments in the article.

### 2.8 Quantitative real-time PCR

Total mRNA was extracted from tissues and cells with Trizol reagent (R401-01, Vazyme, China), quantified by NanoDrop 2000, and reverse-transcribed to cDNA using the RevertAid First Strand cDNA Synthesis kit (K1622, Thermo Fisher Scientific, USA) according to the manufacturer’s protocol. Real-time PCR was performed using LightCycler96 (Roche, Switzerland). Melting curve analysis was performed at the end of each PCR reaction. The sequences of oligonucleotide primers are listed in **Additional file 1:** Table S2. All mRNA measurements were calculated by the 2^‐ΔΔct^ method and normalized to *18S* mRNA levels.

### 2.9 Co-immunoprecipitation assay and ubiquitylation analysis

To determine the mode of Smad7 degradation, the inhibitor of protein synthesis in eukaryotes, cycloheximide (CHX; 10 μmol/L; HY-12320, Med Chem Express, USA), the proteasome inhibitor MG132 (10 μmol/L; HY-13259, Med Chem Express, USA), and the lysosome inhibitor chloroquine (10 μmol/L; HY-17589A, Med Chem Express, USA) were used. To determine the interaction between proteins, the cells were lysed with lysis buffer. After 30 min of IP lysis, the samples were centrifuged for 15 min at 12,000× *g* at 4 °C to remove debris, and aliquots of the supernatants were assayed for total protein content by the BCA method (23225, Thermo Fisher Scientific, USA). One part was used for input, and the others for IP. For every 1000 μg of total protein, 1 μg of anti-Smad7 antibody (ab272928, Abcam, UK) was added, and the mixture was incubated at 4 °C with rotation. Subsequently, 40 μl of protein A/G agarose (HY-K0202, Med Chem Express, USA) was introduced, and after a 2‑hour incubation, the samples were further incubated overnight at 4 °C. The next day, magnetic beads were removed with DynaMag (Thermo Fisher Scientific, USA). The input and IP samples were subjected to Western blotting experiments.

Other detailed methods are provided in **Additional file 1: Methods**.

### 2.10 Statistical analysis

All data were analyzed with ImageJ v1.53e and GraphPad Prism v10.1.2, and data are presented as mean±standard error of the mean (SEM). Unpaired two-tailed Student’s *t*-test was used for comparisons between two groups. One-way ANOVA or two-way ANOVA was employed for multiple comparisons. Statistical significance was considered for *P*-values below 0.05, with *P<0.05, **P<0.01, and ***P<0.001. The exact sample size (*n*) is reported in the figure legends.

## 3 Results

### 3.1 Deletion of the *EP3* gene improves ISO-induced cardiac diastolic dysfunction and fibrosis

To determine whether EP3 participates in ISO-induced cardiac remodeling, we treated the WT mice with ISO [5 mg/(kg·d)] subcutaneously for 7 d to establish an ISO‑induced cardiac fibrosis model, evidenced by expression of *α-SMA*, *collagen III*, and *EP3* were markedly upregulated at mRNA and protein levels (**Additional file 1:** Fig. S1a**, b**). Furthermore, immunofluorescence staining revealed that EP3 was predominantly expressed in CFs and its levels were significantly increased following ISO treatment (**Additional file 1:** Fig. S1c).

Additionally, Western blotting analysis of cultured primary CMs and CFs from neonatal rats showed that EP3 was abundantly expressed in CFs, whereas its expression in CMs was much lower (**Additional file 1:** Fig. S1d). This finding suggests that CF-EP3 may be involved in ISO-induced cardiac fibrosis.

Then, we used the previously generated EP3^−/−^ mice and their WT littermates to establish ISO-induced cardiac fibrosis (Fig. 1**a**). Successful *EP3* gene deletion in EP3^−/−^ mice was confirmed by the reduced *EP3* mRNA expression in heart tissue (**Additional file 1:** Fig. S2a**-c**). The results showed that the cardiac diastolic function declined in WT mice following 7 d of ISO injection, which was significantly improved in the EP3^−/−^ mice, as indicated by the reversed early-to-atrial wave ratio (E/A ratio) (Fig. 1**b**). ISO-induced significantly increased heart weight (HW, mg) to body weight (BW, g) ratio (HW/BW ratio), the HW (mg) to tibia length (TL, mm) ratio (HW/TL ratio), and left ventricular mass (LV mass) compared with saline in both WT and EP3^−/−^ mice, whereas left ventricular anterior wall thickness (LVAW) and left ventricular posterior wall thickness (LVPW) were not significantly altered. *EP3* gene deletion does not affect cardiac hypertrophy. Additionally, the assessment of ejection fraction (EF), fractional shortening (FS), left ventricular internal diameter (LVID), and left ventricular volume (LV Vol) and heart rate showed no significant in systolic function between WT and EP3^−/−^ mice (**Additional file 1:** Fig. S3). Masson’s trichrome staining and Sirius Red staining the panoramic view (PV) of the heart revealed a significant increase in ECM deposition in ISO-treated WT mice, which was predominantly localized to the papillary muscle (PM) and interstitium (IS), rather than to the surrounding area of the blood vessel (BV). In contrast, the deletion of the *EP3* gene markedly attenuated ISO-induced ECM deposition (Fig. 1**c, d**). Western blotting analysis revealed that the ISO‑induced increases in α‑SMA and collagen III were also significantly reduced in EP3^−/−^ mice (Fig. 1**e**), and immunofluorescence staining further confirmed the reduction of collagen III (Fig. 1**f**).

**Fig. 1:**
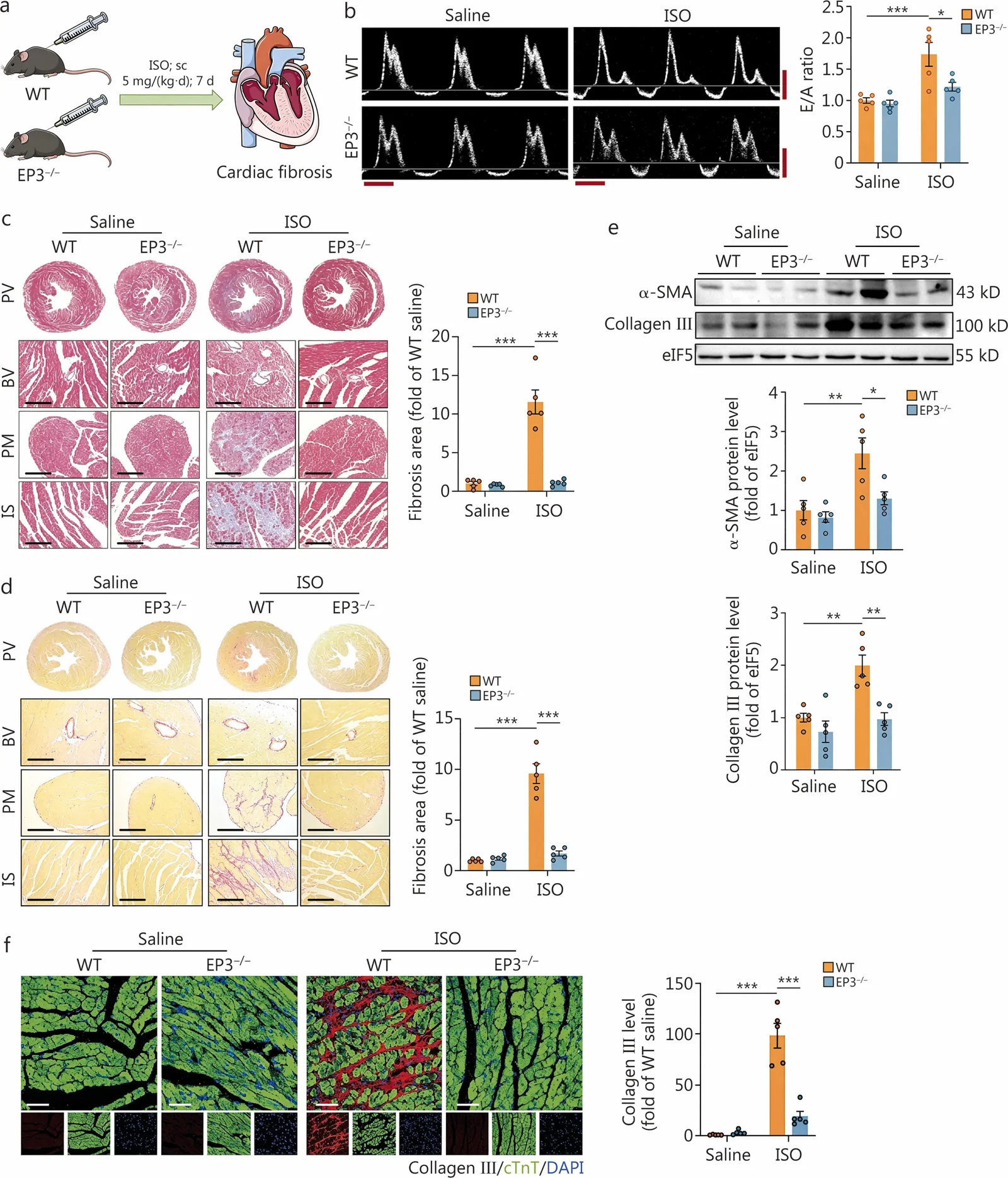
Global knockout of the *EP3* gene attenuates ISO-induced cardiac diastolic dysfunction and fibrosis. **a** The experimental protocol involved administering a 7-day course of subcutaneous injections of ISO 5 mg/(kg·d) to both WT and EP3^−/−^ mice to induce cardiac fibrosis. **b** The Doppler color ultrasound measurements of the peak velocity flow in early diastole (E, m/s) and late diastole by atrial contraction (A, m/s) ratio (E/A ratio) in WT and EP3^−/−^ mice injected with saline or ISO for 7 d. The horizontal scale bar represents a time course of 100 ms. The vertical scale represents a blood flow velocity of 4 m/s. *n*=5. Masson trichrome staining (**c**) and Sirius Red staining (**d**) of the ventricular sections of WT and EP3^−/−^ mice treated with saline or ISO for 7 d. Scale bar=100 μm. *n*=5. **e** Western blotting analysis of α-SMA and collagen III protein levels in the ventricles of the WT and EP3^−/−^ mice treated with saline or ISO for 7 d, with eIF5 as an internal control. *n*=5. **f** Immunofluorescence staining of collagen III (in red) and cTnT (in green) in the ventricular sections of the WT and EP3^−/−^ mice treated with saline or ISO for 7 d, DAPI stained the nuclei (in blue). Scale bar=50 μm. *n*=5. Data were presented as mean±SEM, *P<0.05, **P<0.01, ***P<0.001. SEM. Standard error of the mean; WT. Wild-type; EP3. E-type prostaglandin receptor 3; ISO. Isoproterenol; sc. Subcutaneous injection; α-SMA. α-smooth muscle actin; eIF5. Eukaryotic translation initiation factor 5; cTnT. Cardiac troponin I; DAPI. 4',6-diamidino-2-phenylindole. PV. Panoramic view; BV. Blood vessels; PM. Papillary muscle; IS. Interstitium.

Together, these findings demonstrate that global deletion of the *EP3* gene improves ISO-induced cardiac diastolic dysfunction and fibrosis, but has no effect on cardiac hypertrophy and left ventricular systolic function.

### 3.2 Deletion of CF-*EP3* ameliorates ISO-induced cardiac fibrotic remodeling

To further investigate whether the deletion of the CF-*EP3* ameliorates ISO-induced cardiac fibrosis and diastolic dysfunction, we generated EP3^cKO^ by crossing the EP3^f/f^ mice with S100A4^Cre+^ mice (**Additional file 1:** Fig. S4a). Successful CF-*EP3* deletion in EP3^cKO^ mice was confirmed by the expression of the *EP3* floxed, WT allele and S100A4^Cre+^ in tail, the upper panel shows the floxed *EP3* allele (194 bp) and WT allele (160 bp), and the lower panel shows the S100A4^Cre+^ (377 bp) (**Additional file 1:** Fig. S4b), recombined floxed *EP3* allele and S100A4^Cre+^ in heart, the upper panel shows the recombined floxed *EP3* allele (229 bp) and the lower panel shows the S100A4^Cre+^ (377 bp) (**Additional file 1:** Fig. S4c), and the reduction of *EP3* mRNA levels (**Additional file 1:** Fig. S4d). Both the control littermates (EP3^f/f^) and EP3^cKO^ mice were subcutaneously administered with ISO for 7 d (Fig. 2**a**). Consistent with the observations in the EP3^−/−^ mice, the cardiac diastolic function was improved in the EP3^cKO^ mice as indicated by the decreased E/A ratio (Fig. 2**b**). However, the knockout of the *EP3* gene in the fibroblasts had no effect on cardiac hypertrophy and left ventricular systolic function (**Additional file 1:** Fig. S5).

**Fig. 2:**
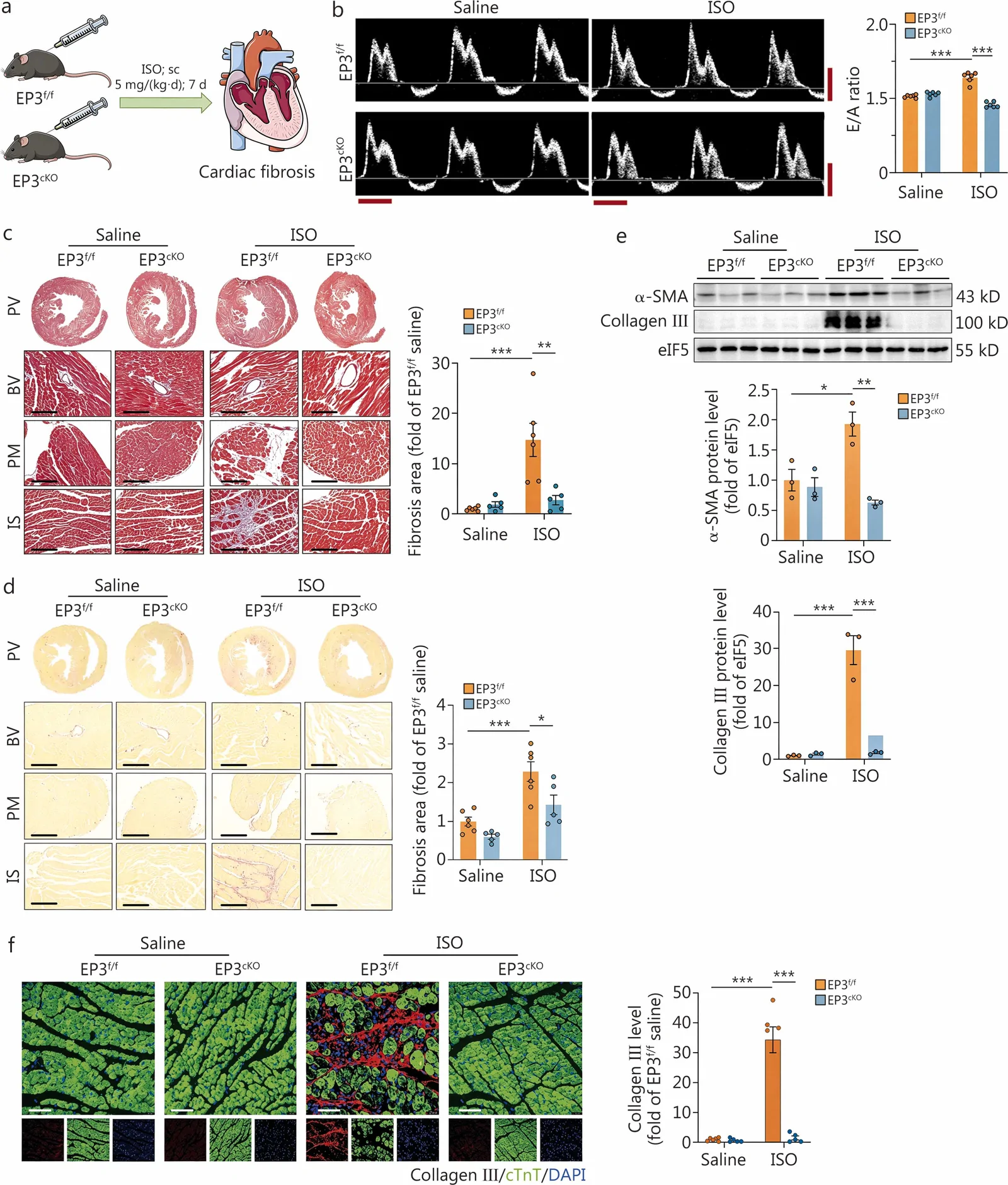
Cardiac fibroblast (CF)-specific deletion of the *EP3* gene (EP3^cKO^) attenuates ISO-induced cardiac diastolic dysfunction and fibrosis. **a** The experimental scheme showing the EP3^f/f^ and EP3^cKO^ mice receiving subcutaneous injection of ISO 5 mg/(kg·d) for 7 d to induce cardiac fibrosis. **b** The Doppler color ultrasound measurements of the peak velocity flow in early diastole (E, m/s) and late diastole by atrial contraction (A, m/s) ratio (E/A ratio) in EP3^f/f^ and EP3^cKO^ mice injected with saline or ISO for 7 d. The horizontal scale bar represents a time course of 100 ms. The vertical scale represents a blood flow velocity of 4 m/s. *n*=6. Masson trichrome staining (**c**) and Sirius Red staining (**d**) of the ventricular sections of EP3^f/f^ and EP3^cKO^ mice treated with saline or ISO for 7 d. Scale bar=100 μm. *n*=5−6. **e** Western blotting analysis of α-SMA and collagen III protein levels in the ventricles of the EP3^f/f^ and EP3^cKO^ mice treated with saline or ISO for 7 d, eIF5 was used as an internal control. *n*=3. **f** Immunofluorescence staining of collagen III (in red) and cTnT (in green) in the ventricular sections of the EP3^f/f^ and EP3^cKO^ mice treated with saline or ISO for 7 d, DAPI stained the nuclei (in blue). Scale bar=50 μm. *n*=5−6. Data were presented as mean±SEM, *P<0.05, **P<0.01, ***P<0.001. SEM. Standard error of the mean; EP3. E-type prostaglandin receptor 3; ISO. Isoproterenol; sc. Subcutaneous injection; α-SMA. α-smooth muscle actin; eIF5. Eukaryotic translation initiation factor 5; cTnT. Cardiac troponin I; DAPI. 4’,6-diamidino-2-phenylindole. PV. Panoramic view; BV. Blood vessels; PM. Papillary muscle; IS. Interstitium.

As anticipated, compared with the EP3^f/f^ mice, there was minimal ECM deposition in the PV of the heart, primarily in the PM and IS regions of the EP3^cKO^ mice following ISO treatment. Little ECM was deposited in the surrounding area of the BV, and the amounts were similar between the two groups, as demonstrated by Masson trichrome staining and Sirius Red staining (Fig. 2**c, d**). Consistently, Western blotting revealed that the ISO‑induced increases in α‑SMA and collagen III were significantly attenuated in EP3^cKO^ mice compared with EP3^f/f^ mice (Fig. 2**e**). Immunofluorescence staining further confirmed the reduction of collagen III (Fig. 2**f**), and immunohistochemistry also validated the decreases in both α‑SMA and collagen III (**Additional file 1:** Fig. S6).

Interestingly, CM-specific *EP3* knockout mice (EP3^mKO^) do not affect ISO-induced cardiac fibrosis and diastolic dysfunction (**Additional file 1:** Fig. S7). Additionally, deletion of the CM-*EP3* had no effect on cardiac hypertrophy and systolic function in mice (**Additional file 1:** Fig. S8). Together, these results demonstrate that specific deletion of the *EP3* gene in the CFs, rather than in the CMs, mitigates ISO-induced cardiac diastolic dysfunction and fibrosis.

### 3.3 Blockade of the EP3 in the CFs suppresses TGF-β1-induced activation and ECM production

It is well established that upon β-AR activation, the CMs release pro-fibrotic cytokines, especially TGF-β1, which in turn stimulate the CFs to undergo transdifferentiation and produce ECM, thereby driving the fibrotic process, as demonstrated in ISO-induced cardiac fibrosis models [6], [28], [29], [30]. To this end, primary CMs and CFs were isolated from neonatal rat hearts; CMs were treated with ISO to obtain conditioned medium (CM-ISO), which were then used to stimulate CFs which were pretreated with DG041 or L798106, followed by assessment of fibrotic marker expression (Fig. 3**a**). The results showed that the CM-ISO led to a significant increase in α-SMA and collagen III levels in the CFs, which was abolished by pretreatment with EP3 antagonists (DG041 or L798106) (Fig. 3**b, c**).

**Fig. 3:**
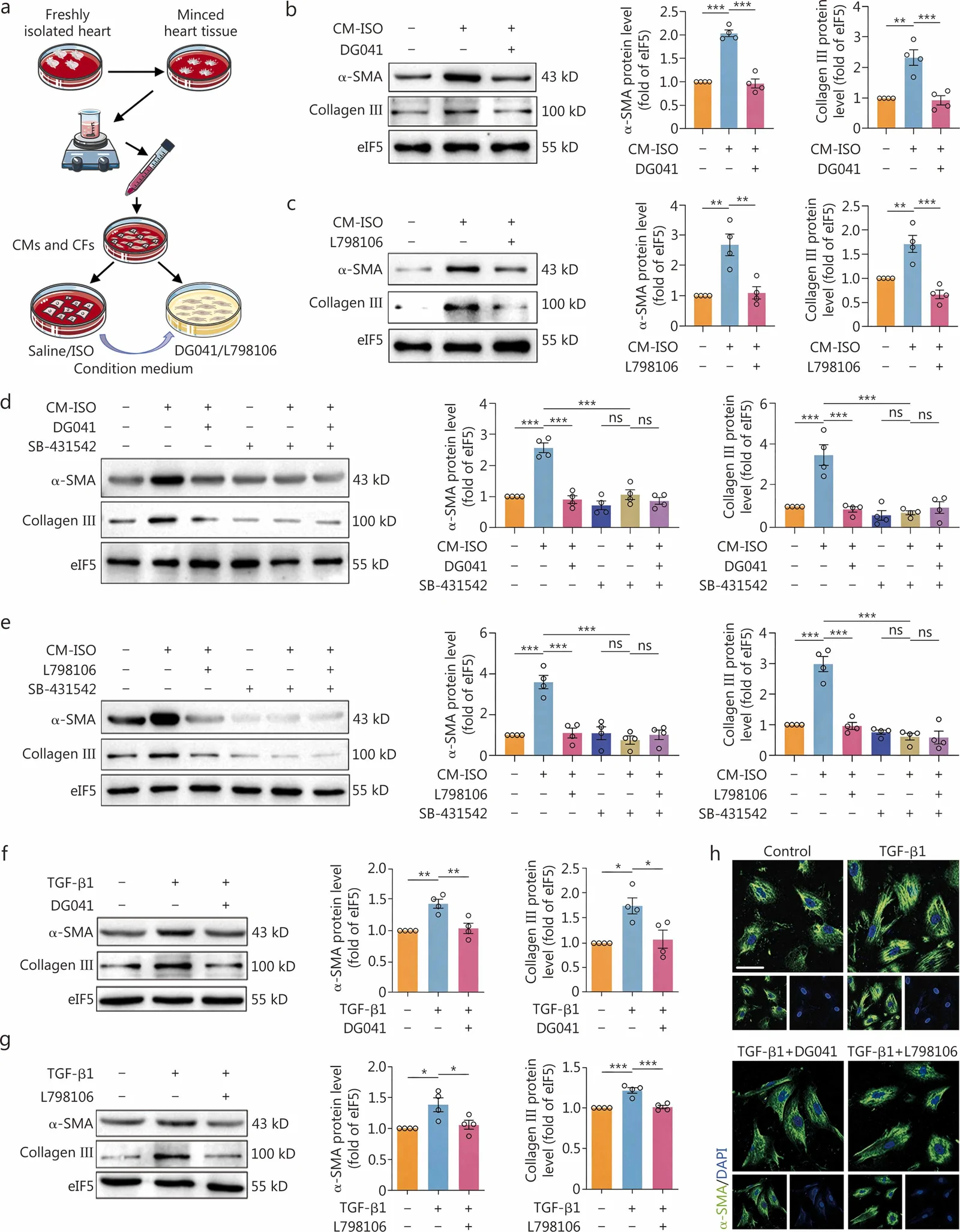
Blockade of cardiac fibroblast (CF)-EP3 suppresses TGF-β1-mediated CF activation and ECM production. **a** After the CMs were treated with ISO for 24 h, the supernatants were collected as conditioned medium to treat the CFs. **b** The protein expression of α-SMA and collagen III after pretreatment with DG041 (1 μmol/L) for 30 min and then incubated with saline or ISO-treated CM-derived conditioned medium for another 24 h in CFs. eIF5 was used as an internal control. *n*=4. **c** The protein expression of α-SMA and collagen III after pretreatment with L798106 (1 μmol/L) for 30 min and then incubated with saline or ISO-treated CM-derived conditioned medium for another 24 h in CFs. eIF5 was used as an internal control. *n*=4. **d** The protein expression of α-SMA and collagen III after pretreatment with 30 min pretreatment of SB-431542 (10 μmol/L) and then treated with DG041 (1 μmol/L) for 30 min, followed by incubation with saline or ISO-treated CM-derived conditioned medium for another 24 h. eIF5 was used as an internal control. *n*=4. **e** The protein expression of α-SMA and collagen III after pretreatment with 30 min pretreatment of SB-431542 (10 μmol/L) and then treated with L798106 (1 μmol/L) for 30 min, followed by incubation with saline- or ISO-treated CM-derived conditioned medium for another 24 h. eIF5 was used as an internal control. *n*=4. **f** The protein expression of α-SMA and collagen III after DG041 (1 μmol/L) for 30 min and then treated with TGF-β1 (10 ng/ml) for another 24 h in CFs. eIF5 was used as an internal control. *n*=4. **g** The protein expression of α-SMA and collagen III after pretreatment with L798106 (1 μmol/L) for 30 min and then treated with TGF-β1 (10 ng/ml) for another 24 h in CFs. eIF5 was used as an internal control. *n*=4. **h** Immunofluorescence staining showing α-SMA expression (in green) after 24-hour TGF-β1 (10 ng/ml) treatment in the CFs pretreated with DG041 or L798106 for 30 min. DAPI stains the nuclei (in blue). Scale bar=50 μm. Data were presented as mean±SEM, *P<0.05, **P<0.01, ***P<0.001, ns non-significant. SEM. Standard error of the mean; CM-ISO. Conditioned medium of CMs treated with ISO; α-SMA. α-smooth muscle actin; eIF5. Eukaryotic translation initiation factor 5; TGF-β1. Transforming growth factor-β1; DAPI. 4',6-diamidino-2-phenylindole; CMs. Cardiomyocytes; ECM. Extracellular matrix.

To investigate whether EP3 affects TGF-β1-induced CFs activation, we pretreated with SB-431542, a specific TGF-β receptor kinase inhibitor, prior to CM‑ISO stimulation. This pretreatment eliminated the increase in α-SMA and collagen III expression triggered by the conditioned medium. Furthermore, the EP3 antagonists (DG041 or L798106) had no additional inhibitory effect on the expression of α-SMA and collagen III (Fig. 3**d, e**). This indicates that blocking EP3 primarily inhibits the activation of the CFs induced by CM-derived TGF-β1. To further support this conclusion, we conducted experiments using a human recombinant TGF-β1 protein to treat the CFs. As expected, the Western blotting results showed that both DG041 and L798106 pretreatment significantly abolished TGF-β1-elicited α-SMA and collagen III expression (Fig. 3**f, g**). In addition, immunofluorescence staining of α-SMA also revealed that blockade of EP3 by DG041 or L798106 suppressed α-SMA upregulation induced by TGF-β1 (Fig. 3**h**). Conversely, activation of EP3 by sulprostone, a selective EP3 agonist, enhanced TGF-β1-induced CF activation, as evidenced by a significant increase in α-SMA and collagen III expression (**Additional file 1:** Fig. S9). Taken together, these data demonstrate that blockade of EP3 suppresses TGF-β1-induced CF activation and ECM production. In addition, blockade of EP3 failed to suppress ISO-induced increase of TGF-β1 in the CMs (**Additional file 1:** Fig. S10a**, b**). However, we found that administration of TGF-β1 induced the transformation of CFs into myofibroblasts, as evidenced by the upregulation of α-SMA, which was accompanied by the upregulation of EP3 in the CFs (**Additional file 1:** Fig. S10c). These findings suggest that upon ISO treatment, the CMs release TGF-β1, and then target the CFs to increase EP3 expression *in vivo*.

### 3.4 Inhibition of EP3 suppresses TGF-β1-elicited Smad2/3 phosphorylation by up-regulating Smad7 protein level

To further investigate the mechanism by which EP3 enhances TGF-β1-induced CF activation, we firstly examined the phosphorylation levels of Smad2 and Smad3, which are the primary effectors of TGF-β1 that exert the pro-fibrotic functions. The results showed that pretreatment with either DG041 or L798106 strongly suppressed TGF-β1-provoked phosphorylation of Smad2 and Smad3 (Fig. 4**a, b**). Furthermore, immunofluorescence staining revealed that inhibiting EP3 with DG041 reduced, whereas activating EP3 with sulprostone enhanced, the nuclear translocation of Smad3 triggered by TGF-β1 (Fig. 4**c**). It is well established that the phosphorylation of Smad2 and Smad3 is negatively regulated by the inhibitory Smad7 [31]. To explore this further, we examined Smad7 expression in the CFs following treatment with the EP3 antagonists (DG041 or L798106). The results showed that while the administration of EP3 antagonists had no significant effect on *Smad7* mRNA levels (**Additional file 1:** Fig. S11), it notably increased Smad7 protein expression in 3 h (Fig. 4**d, e**). In contrast, treatment with sulprostone remarkably decreased Smad7 expression in a dose- and time-dependent manner in CFs (**Additional file 1:** Fig. S12). These results suggest that EP3 suppresses Smad7 at the post-transcriptional level. We therefore investigated whether EP3 inhibits Smad7 protein expression through suppressed protein synthesis or via the ubiquitin-proteasome and lysosome-dependent degradation pathways. Our findings revealed that, in the presence of the translation inhibitor CHX, the EP3 antagonists also led to an upregulation of Smad7 (Fig. 4**f, g**), indicating that EP3 does not regulate Smad7 protein expression at the translational level. Furthermore, we found that, in the presence of the proteasome inhibitor MG132, rather than the lysosome inhibitor chloroquine, the EP3 agonist no longer decreased Smad7 protein expression (Fig. 4**h, i**), suggesting that EP3 decreased Smad7 protein levels via the ubiquitin-proteasome-dependent degradation pathway.

**Fig. 4:**
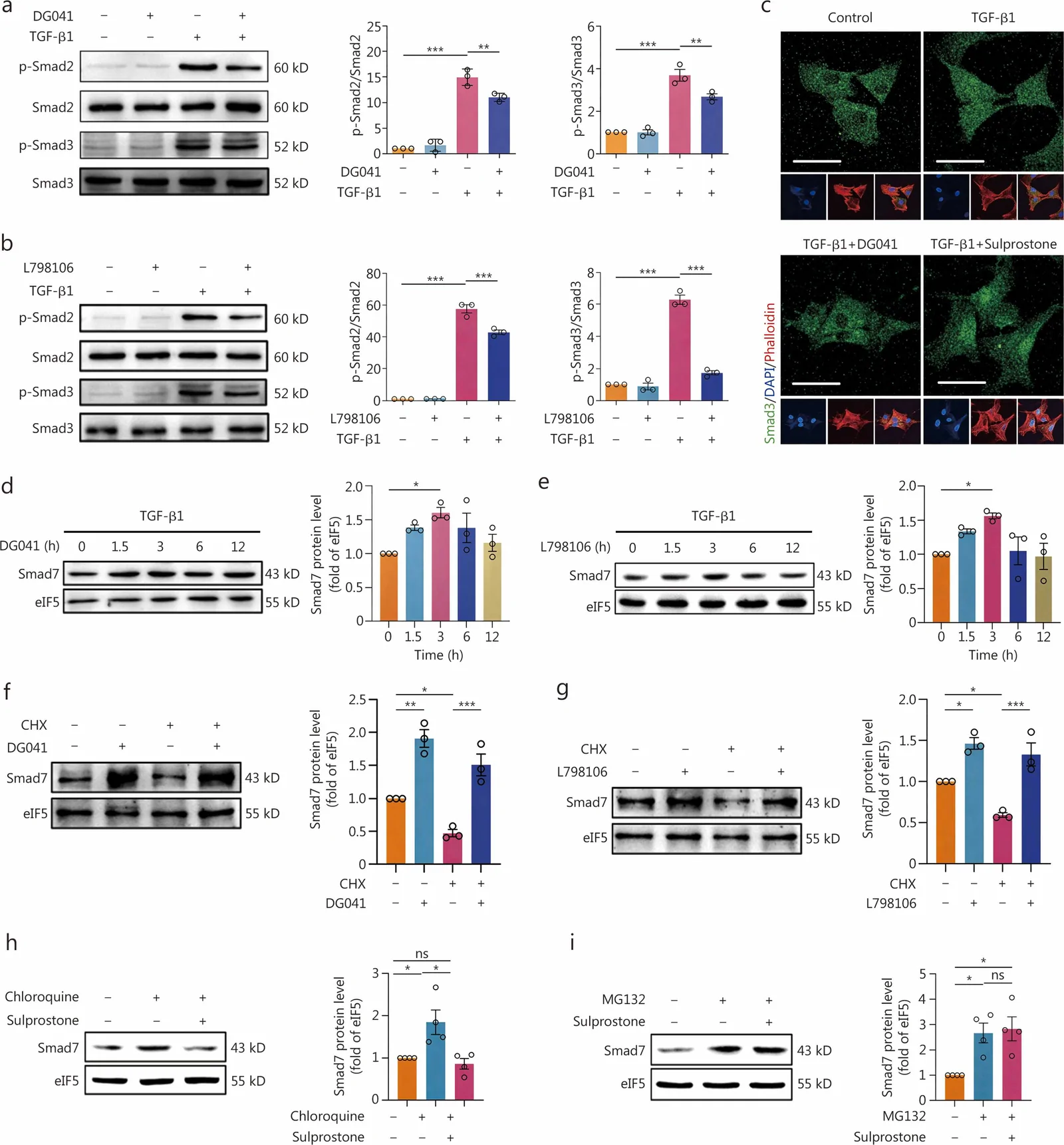
Inhibition of EP3 suppresses TGF-β1-elicited Smad2/3 activation by up-regulating Smad7 protein level in the CFs. **a** The protein expression of phosphorylated Smad2 (p-Smad2), total Smad2 (Smad2), phosphorylated Smad3 (p-Smad3), and total Smad3 (Smad3) after pretreatment of DG041 (1 μmol/L) for 30 min and then treated with TGF-β1 (10 ng/ml) for another 3 h in CFs. *n*=3. **b** The protein expression of phosphorylated Smad2 (p-Smad2), total Smad2 (Smad2), phosphorylated Smad3 (p-Smad3), and total Smad3 (Smad3) after pretreatment with L798106 (1 μmol/L) for 30 min and then treated with TGF-β1 (10 ng/ml) for another 3 h in CFs. *n*=3. **c** Immunofluorescence staining showing the effects of DG041 (1 μmol/L) or L798106 (1 μmol/L) on TGF-β1 (10 ng/ml)-induced Smad3 nuclear translocation (in green). DAPI stains the nuclei (in blue). Scale bar=50 μm. **d** The protein expression of Smad7 treated with DG041 (1 μmol/L) for different time periods (0, 1.5, 3, 6, 12 h) in the presence of TGF-β1 (10 ng/ml). *n*=3. **e**. The protein expression of Smad7 treated with L798106 (1 μmol/L) for different time periods (0, 1.5, 3, 6, 12 h) in the presence of TGF-β1 (10 ng/ml). *n*=3. **f** The protein expression of Smad7 after pretreatment with CHX (10 μmol/L) for 30 min, followed by treatment with DG041 (1 μmol/L) for 3 h. eIF5 was used as an internal control. *n*=3. **g** The protein expression of Smad7 after pretreatment with CHX (10 μmol/L) for 30 min, followed by treatment with L798106 (1μmol/L) for 3 h. eIF5 was used as an internal control. *n*=3. **h** The protein expression of Smad7 after pretreatment with chloroquine (10 μmol/L) for 30 min and followed by treatment with sulprostone (1 μmol/L) for 3 h, with eIF5 as an internal control. *n*=4. **i** The protein expression of Smad7 after pretreatment with MG132 (10 μmol/L) for 30 min and followed by treatment with sulprostone (1 μmol/L) for 3 h, with eIF5 as an internal control. *n*=4. Data were presented as mean±SEM, *P<0.05, **P<0.01, ***P<0.001, ns non-significant. SEM. Standard error of the mean; TGF-β1. Transforming growth factor-β1; Smad. Small mother against decapentaplegic; CHX. Cycloheximide; EP3. E-type prostaglandin receptor 3; CFs. Cardiac fibroblasts; eIF5. Eukaryotic translation initiation factor 5.

### 3.5 Blockade of *EP3* reduces Smad7 ubiquitination through decreasing Smurf2 expression in a phosphatidylinositol 3-kinase (PI3K)/protein kinase B (Akt)-dependent manner

It has been reported that Smad7 protein expression is regulated by Smurf2 (an E3 ubiquitin ligase), which promotes the degradation of Smad7 protein [32]. Then we examined the role of EP3 in Smurf2 expression in the CFs. The results showed that both DG041 and L798106 induced a marked increase in Smad7 protein levels, accompanied by a decrease in Smurf2 protein levels, under both TGF‑β1‑treated and untreated conditions (Fig. 5**a, b**). Additionally, a co-immunoprecipitation (Co-IP) assay using a Smad7 primary antibody revealed that sulprostone markedly increased the ubiquitination of Smad7 protein (Fig. 5**c**; **Additional file 1:** Fig. S13). Next, we investigated whether the inhibition of EP3 suppresses the ubiquitination of Smad7 protein in the ubiquitin-overexpressing CFs. With the decrease of Smurf2 in the whole cell lysate, the Co-IP results showed that both DG041 and L798106 reduced the ubiquitination of Smad7 protein (Fig. 5**d, e**).

**Fig. 5:**
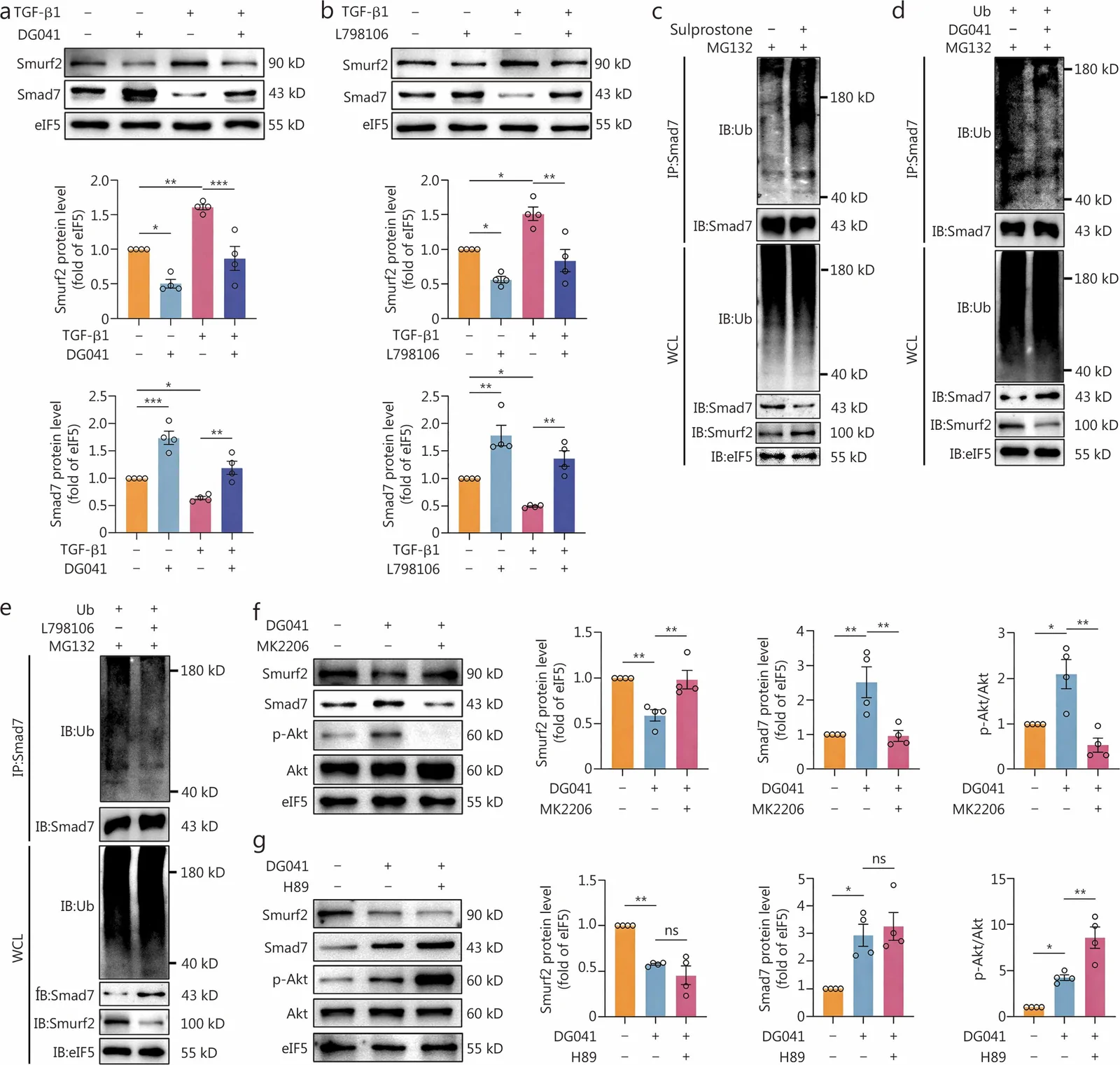
Blockade of EP3 reduces Smad7 ubiquitination through decreasing Smurf2 expression in the CFs. **a** The protein expression of Smurf2 and Smad7 after pretreatment with DG041 (1 μmol/L) for 30 min and then treated with TGF-β1 (10 ng/ml) for 3 h, with eIF5 as an internal control. *n*=4. **b** The protein expression of Smurf2 and Smad7 after pretreatment with L798106 (1 μmol/L) for 30 min and then treated with TGF-β1 (10 ng/ml) for 3 h, with eIF5 as an internal control. *n*=4. **c** The CFs were pretreated with sulprostone (1 μmol/L) for 30 min and followed by the treatment of MG132 (10 μmol/L) for 3 h. Co-immunoprecipitation (Co-IP) was performed using the rabbit anti-Smad7 primary antibody, and the immunoprecipitated proteins were blotted with an anti-ubiquitin primary antibody. The whole cell lysate (WCL) was used to examine the total protein levels of ubiquitin, Smad7, and Smurf2. eIF5 was used as an internal control. The CFs were transfected with a ubiquitin overexpressing vector for 36 h and then pretreated with DG041 (1 μmol/L) (**d**) or L798106 (1 μmol/L) (**e**) for 30 min, followed by treatment with MG132 (10 μmol/L) for another 3 h. Co-IP was performed using the rabbit anti-Smad7 primary antibody, and the immunoprecipitated proteins were blotted with an anti-ubiquitin primary antibody. The WCL was used to examine the total protein levels of ubiquitin, Smad7, and Smurf2; eIF5 was used as an internal control. **f** The protein expression of Smurf2, Smad7, phosphorylated Akt (p-Akt), and total Akt (Akt) after pretreatment with MK2206 (5 μmol/L) for 30 min and then treated with DG041 (1 μmol/L) for 3 h, with eIF5 as an internal control. *n*=4. **g** The protein expression of Smurf2, Smad7, p-Akt, and Akt after pretreatment of H89 (10 μmol/L) for 30 min and then treated with DG041 (1 μmol/L) for 3 h, with eIF5 as an internal control. *n*=4. Data were presented as mean±SEM, *P<0.05, **P<0.01, ***P<0.001, ns non-significant. SEM. Standard error of the mean; TGF-β1. Transforming growth factor-β1; Smad. Small mother against decapentaplegic; Smurf2. Smad ubiquitination regulatory factor 2; eIF5. Eukaryotic translation initiation factor 5; Ub. Ubiquitin; Akt. Protein kinase B; EP3. E-type prostaglandin receptor 3; CFs. Cardiac fibroblasts.

Akt is a kinase capable of phosphorylating Smurf2, leading to a reduction of Smurf2 protein levels through ubiquitin/proteasome-mediated degradation [33]. We hypothesize that blocking EP3 may elevate intracellular cAMP levels, thereby activating the PI3K/Akt pathway, which is expected to result in the downregulation of Smurf2 and the upregulation of Smad7 [34]. Notably, our findings indicate that treatment with both DG041 and L798106 increased intracellular cAMP levels and promoted Akt phosphorylation, accompanied by a reduction in Smurf2 expression and an elevation in Smad7 expression (Fig. 5**f**; **Additional file 1:** Fig. S14a**, b**). Moreover, pretreatment with MK2206, a selective Akt inhibitor, blocked DG041- and L798106-induced reduction in Smurf2 and increase in Smad7 levels, with intracellular cAMP levels unaffected (Fig. 5**f**; **Additional file 1:** Fig. S14a**, b**). Additionally, H89, a selective protein kinase A (PKA) inhibitor, exhibited little effect on the DG041- and L798106-mediated reduction in Smurf2 and increase in Smad7 expression, as well as intracellular cAMP levels (Fig. 5**g**; **Additional file 1:** Fig. S14c**, d**). These findings collectively suggest that blocking EP3 activates the PI3K/Akt pathway, leading to a decrease in Smurf2 levels and a reduction in Smad7 ubiquitination, which subsequently increases Smad7 protein levels.

### 3.6 Pharmacological blockade of EP3 improves ISO-induced cardiac fibrosis and diastolic dysfunction

To test whether pharmacological inhibition of EP3 improves ISO-induced cardiac fibrosis *in vivo*, we pretreated mice with the EP3 antagonist DG041 for 1 d prior to ISO injection, followed by co-administration of DG041 and ISO for 7 d (Fig. 6**a**). As expected, echocardiographic measurement revealed that DG041 treatment significantly improved cardiac diastolic function as revealed by decreased E/A ratio (Fig. 6**b**). Moreover, pharmacological blockade of EP3 had no significant effect on cardiac hypertrophy and left ventricular systolic function (**Additional file 1:** Fig. S15). Masson trichrome staining and Sirius Red staining showed that ECM accumulation in the PM and IS areas was significantly decreased in DG041-treated mice receiving ISO injection (Fig. 6**c, d**). Consistently, DG041 treatment significantly reduced ISO-induced α-SMA and collagen III expression in the heart (Fig. 6**e, f**; **Additional file 1:** Fig. S16). Taken together, these findings demonstrate that pharmacological blockade of EP3 improves ISO-induced cardiac fibrosis.

**Fig. 6:**
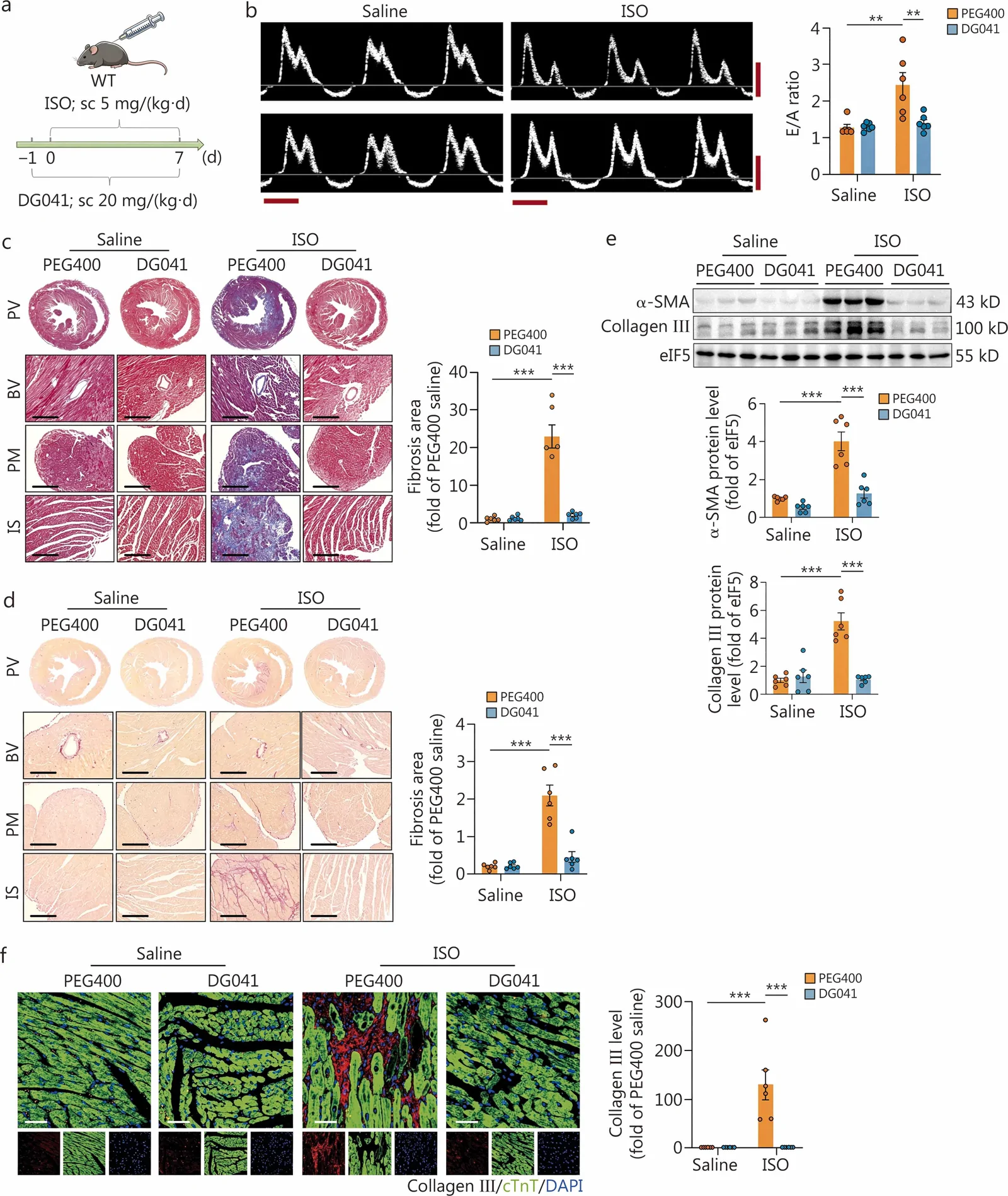
Pharmacological blockade of EP3 attenuates ISO-induced cardiac diastolic dysfunction and fibrosis. **a** The experimental scheme showing that WT mice were injected with DG041 [20 mg/(kg∙d), subcutaneously (sc)] or PEG400 (the vehicle of DG041) from day −1 to day 7, and ISO [5 mg/(kg∙d), sc] or saline was injected from day 0 to day 7 to evaluate the effect of pharmacological inhibition of EP3 on ISO-induced cardiac fibrosis. **b** The Doppler color ultrasound measurements of the peak velocity flow in early diastole (E, m/s) and late diastole by atrial contraction (A, m/s) ratio (E/A ratio) in the PEG400- or DG041-treated mice injected with saline or ISO for 7 d. The horizontal scale bar represents a time course of 100 ms. The vertical scale represents a blood flow velocity of 4 m/s. *n*=6. Masson trichrome staining (**c**) and Sirius Red staining(**d**) of the ventricular sections of the PEG400- or DG041-treated mice injected with saline or ISO for 7 d. Scale bar=100 μm. *n*=6. **e** The protein expression of α-SMA and collagen III in the ventricles of the PEG400- or DG041-treated mice injected with saline or ISO for 7 d, eIF5 was used as an internal control. *n*=6. **f** Immunofluorescence staining of collagen III (in red) and cTnT (in green) in the ventricular sections of the PEG400- or DG041-treated mice injected with saline or ISO for 7 d. DAPI stained the nuclei (in blue). Scale bar=50 μm. *n*=6. Data were presented as mean±SEM, **P<0.01, ***P<0.001. SEM. Standard error of the mean; WT. Wild-type; ISO. Isoproterenol; PV. Panoramic view; BV. Blood vessel; PM. Papillary muscle; IS. Interstitium; α-SMA. α-smooth muscle actin; eIF5. Eukaryotic translation initiation factor 5; cTnT. Cardiac troponin I; DAPI. 4’,6-diamidino-2-phenylindole.

## 4 Discussion

Sustained β-adrenergic activation is a well-established driver of pathological cardiac remodeling, but the signaling pathways and intercellular mechanisms linking β-adrenergic stress to cardiac fibrosis remain incompletely defined. In the present study, global and CF-specific knockout of the *EP3* gene attenuated ISO-induced cardiac fibrosis and left ventricular diastolic dysfunction. In contrast, CM-specific *EP3* deletion did not ameliorate either phenotype and did not affect cardiac hypertrophy or systolic function. Mechanistically, ISO increased TGF-β1 expression in CMs, whereas TGF-β1 increased EP3 expression in CFs, supporting a feed-forward CM-CF interaction. In CFs, EP3 increased Smurf2 abundance and Smurf2-mediated ubiquitination of Smad7, thereby weakening the inhibitory effect of Smad7 on TGF-β1-Smad2/3 signaling and promoting CF activation and ECM production. Conversely, EP3 blockade preserved Smad7, likely through cAMP-PI3K/Akt-dependent downregulation of Smurf2. Pharmacological EP3 blockade with DG041 also reduced fibrosis and improved diastolic function. Collectively, these findings identify CF-expressed EP3 as an amplifier of CM-derived TGF-β1 signaling and define an EP3-Smurf2-Smad7-Smad2/3 axis in ISO-induced cardiac fibrosis (Fig. 7).

**Fig. 7:**
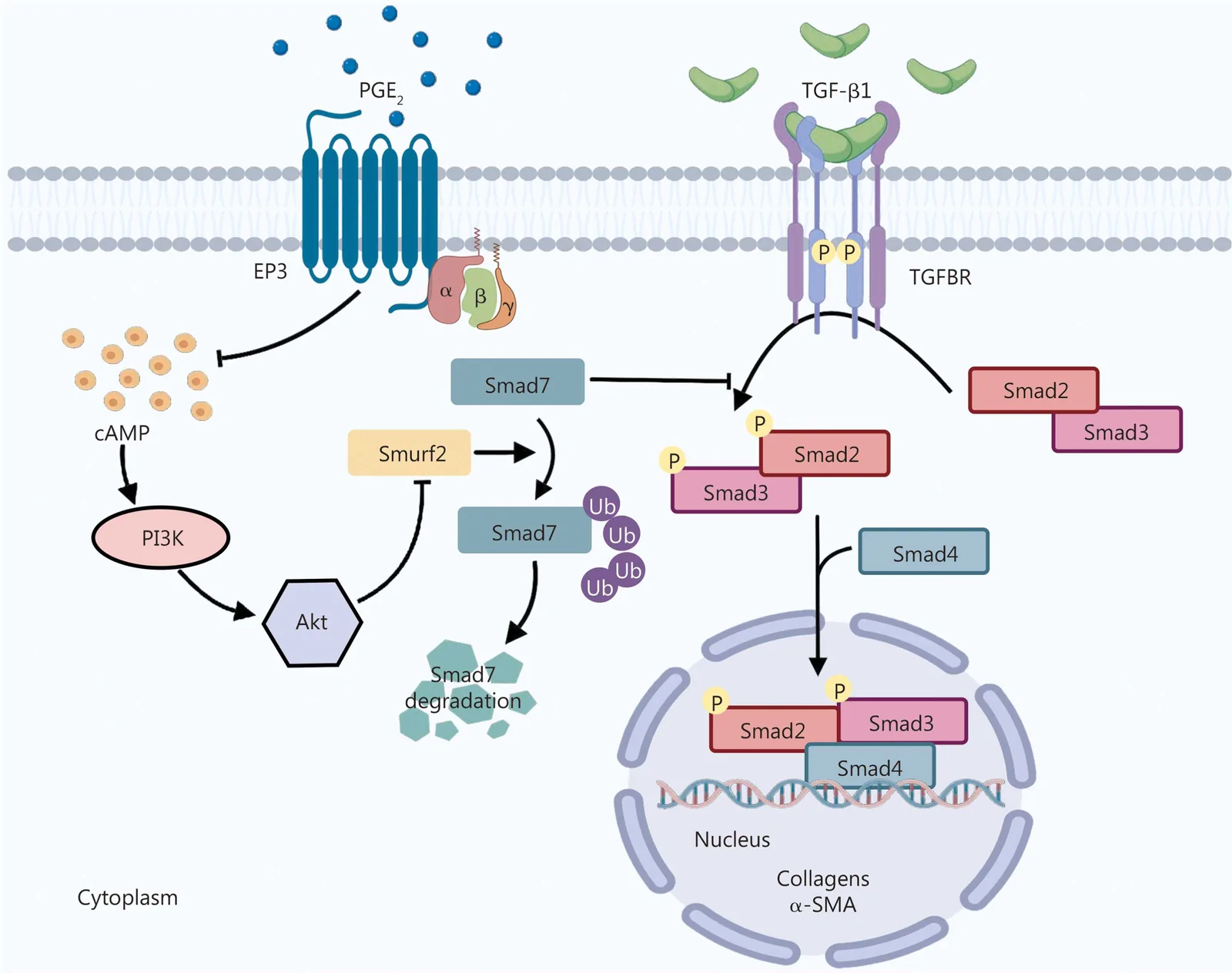
Graphical mechanism showing EP3 inhibits the PI3K/Akt pathway, leading to an increase in Smurf2 levels and an enhancement in Smad7 ubiquitination, which subsequently decreases Smad7 protein levels. This process promotes the phosphorylation of Smad2/3, ultimately activating the activation of cardiac fibroblasts (CFs) and the production of extracellular matrix (ECM). EP3. E-type prostaglandin receptor 3; PGE_2_. Prostaglandin E_2_; cAMP. Cyclic adenosine monophosphate; PI3K. Phosphatidylinositol 3-kinase; Akt. Protein kinase B; Smurf2. Smad specific E3 ubiquitin protein ligase 2; TGF-β1. Transforming growth factor-β1; TGFBR. Transforming growth factor beta receptor; Smad. Small mother against decapentaplegic.

Previous studies have reported divergent effects of PGE_2_ on cardiac remodeling. PGE_2_ was shown to confer cardioprotection by suppressing TGF-β levels, but it was also reported to promote CF proliferation and fibrosis [35], [36]. Our findings are consistent with the latter observation and extend our previous evidence that PGE_2_ promotes cardiac fibrosis through EP4 by identifying a profibrotic role for EP3 in CFs [37]. Notably, EP3 antagonism did not reduce ISO-induced TGF-β1 expression in CMs but attenuated CF responses to the conditioned medium from ISO-treated CMs and exogenous TGF-β1. Thus, unlike previously reported PGE_2_-mediated reduction in TGF-β abundance [35], EP3 primarily regulated fibroblast responsiveness to TGF-β1 in the present model. These apparently divergent effects suggest that the net cardiac action of PGE_2_ depends on the expression and signaling properties of EP1–EP4, the cardiac cell type involved, and the pathological context.

The cell-specific genetic studies further clarify the context-dependent function of cardiac EP3. CM-expressed EP3 has been implicated in regulating cardiac contractility [22]. EP3 overexpression in CMs was found to be associated with impaired cardiac function after myocardial infarction and cardiac hypertrophy; however, EP3 signaling was also shown to protect against myocardial ischemia-reperfusion injury [23], [24], [38]. The absence of a detectable phenotype after CM-specific *EP3* deletion in our study is inconsistent with these reports. The discrepancy may reflect differences in the disease model used, mode of EP3 manipulation, and time point of assessment. By contrast, CF-specific deletion reproduced the protection observed with global *EP3* deletion, consistent with the markedly higher expression of EP3 in CFs than in CMs. Therefore, CFs appear to be the principal site of EP3-mediated profibrotic signaling in the ISO model, although these findings do not exclude a role for CM-EP3 in other cardiac conditions.

The lack of systolic or chronotropic effects should also be interpreted in relation to the ISO protocol. Acute β-AR activation increases cardiac contractility and heart rate [39], and PGE_2_ can acutely inhibit myocardial contraction after β-AR stimulation [40]. In the present study, cardiac function and heart rate were assessed 24 h after the final once-daily ISO injection. Consistent with previous reports, intermittent ISO administration, unlike continuous infusion through an osmotic minipump, caused little persistent change in systolic function or heart rate [41], [42]. *EP3* deficiency likewise did not affect these parameters. Thus, our results do not contradict previous observations of acute β-adrenergic or PGE_2_ actions; rather, they indicate that EP3 predominantly regulates fibrotic remodeling and diastolic dysfunction under the current conditions.

TGF-β is a central regulator of fibrosis and promotes fibroblast-to-myofibroblast transdifferentiation and ECM production mainly through the Smad2/3 signaling [29], [43], [44]. Consistent with reported evidence that inhibition of TGF-β1 or Smad3 alleviates fibrosis [45], EP3 antagonists attenuated TGF-β1-induced Smad2/3 phosphorylation and CF activation. ISO increased TGF-β1 expression in CMs, which was unaffected by EP3 antagonism, possibly because EP3 expression was relatively low in CMs. By contrast, EP3 antagonists inhibited CF activation and ECM production induced by the conditioned medium from ISO-treated CMs, which contained high levels of TGF-β1 [28]. The involvement of TGF-β signaling was further supported by the effects of SB-431542. Moreover, both *in vivo* ISO administration and *in vitro* TGF-β1 exposure increased EP3 expression in CFs. These observations support a feed-forward model in which CM-derived TGF-β1 upregulates EP3 in adjacent CFs, where EP3 subsequently amplifies their responsiveness to TGF-β1. Therefore, EP3 acts downstream of TGF-β1 production in CMs but upstream of sustained Smad2/3 activation in CFs.

Smad7 restrains TGF-β signaling by limiting receptor-dependent Smad2/3 activation, facilitating receptor dephosphorylation or degradation, and inhibiting functional Smad complexes [46], [47]. In the present study, EP3 antagonism was found to increase Smad7 protein abundance and reduce Smad2/3 phosphorylation. These findings extend previous knowledge by linking EP3 signaling to the post-translational regulation of Smad7 and suggest that preservation of Smad7 stability contributes to the antifibrotic effects of EP3 blockade.

To further determine how EP3 regulates Smad7 protein stability, we examined the involvement of the ubiquitin-proteasome pathway using MG132. The results showed that pretreatment of CFs with MG132 prevented the reduction in Smad7 protein induced by the EP3 agonist sulprostone, indicating that EP3-mediated Smad7 loss depends on proteasomal degradation. By contrast, when cells were pre-exposed to sulprostone for 30 min, which allowed EP3 signaling to initiate Smad7 ubiquitination, subsequent MG132 treatment facilitated the detection of the ubiquitinated Smad7 protein by preventing the degradation of all ubiquitinated proteins, a procedure previously reported [48], [49], [50]. These treatment sequences help address distinct but complementary questions, with the former identifying the degradation pathway and the latter capturing the ubiquitination event. Together, these results provide convergent evidence that EP3 destabilizes Smad7 through the ubiquitin-proteasome system.

Smurf2 is conventionally regarded as a negative regulator of TGF-β signaling. Classically, Smad7 recruits Smurf2 to the TGF-β receptor complex, promoting the ubiquitination and degradation of TGF-β type I receptor and receptor-regulated Smads [17], [51]. The present study reveals a novel non-canonical action of Smurf2, through which increased Smurf2 abundance induced by EP3 promotes Smurf2-mediated ubiquitination of Smad7 protein. The resultant loss of Smad7 results in attenuation of its inhibitory effect on the TGF-β pathway, leading to sustained profibrotic Smad2/3 signaling. These findings demonstrate that targeting both EP3 and EP3-induced Smurf2 upregulation holds promise for the treatment of cardiac fibrosis.

Akt-mediated phosphorylation has previously been reported to promote Smurf2 degradation through the ubiquitin-proteasome pathway [33]. In agreement with this observation, our findings support, but do not definitively establish, a model in which EP3 blockade increases intracellular cAMP and activates the PI3K/Akt pathway, thereby reducing Smurf2 levels, stabilizing Smad7 protein, and eventually suppressing Smad2/3 phosphorylation [34]. Conversely, EP3 activation may favor Smurf2 accumulation and Smad7 degradation, thereby weakening the inhibitory effect of Smad7 on TGF-β1-induced CF activation. This mechanism places EP3 upstream of the Smurf2-Smad7 interaction and provides a potential explanation for sustained TGF-β signaling in EP3 activated-CFs.

Our pharmacological findings further support EP3 as a potential intervention target. The beneficial effect of the EP3 antagonist DG041 has been shown in multiple disease models including diet-induced obesity [52], type 2 diabetes [53], and thrombosis [54], [55], [56]. Another EP3 antagonist, L798106, has also been reported to improve myocardial infarction and pulmonary hypertension [25], [57]. However, these EP3 antagonists have not previously been evaluated in cardiac fibrosis. In the present study, we reported, for the first time, that preventive DG041 administration can attenuate ISO-induced fibrosis and improve left ventricular diastolic function. Together with our findings on the beneficial effects of global and CF-specific *EP3* deletion in the ISO-induced animal model, this result provides preclinical proof of concept for EP3 blockade in the treatment of cardiac fibrosis. Nevertheless, it should be mentioned that in our study, since DG041 administration started before ISO exposure, the results suggest that targeting EP3 may represent a novel target for the prevention or early intervention of cardiac fibrosis.

## 5 Limitations

Several limitations should be acknowledged. First, only male mice were used. Whether *EP3* deletion or pharmacological antagonism exerts comparable effects in female mice remains unknown. All *in vivo* findings were obtained using a single, short-term model involving 7 d of ISO exposure. Although this model is suitable for investigating β-adrenergic activation-induced cardiac fibrosis, it does not represent the etiological and temporal heterogeneity of human cardiac fibrosis. In addition, since cardiac function and heart rate were only assessed 24 h after the final ISO injection, the study did not evaluate the acute inotropic and chronotropic responses following each ISO administration.

Second, DG041 was only evaluated at one dose of 20 mg/(kg·d). Treatment began 1 d before the first ISO injection and continued during daily ISO administration for 7 d. Therefore, the present study does not establish a dose-response relationship, minimum effective dose, optimal dose, or therapeutic window. Moreover, because the present study administered DG041 before ISO exposure, whether targeting EP3 is effective in the treatment of established cardiac fibrosis remains unclear and warrants further investigation.

Third, although experiments using the conditioned medium of CM, SB-431542, and exogenous TGF-β1 support a role for CM-derived TGF-β1, conditioned medium may contain additional paracrine factors. The current evidence therefore does not establish TGF-β1 as the sole CM-derived mediator of CF activation. The molecular mechanism by which TGF-β1 upregulates EP3 expression in CFs also remains unclear. Moreover, the precise causal relationships among EP3, cAMP, PI3K/Akt, Smurf2 turnover, and Smad7 ubiquitination, as well as the molecular determinants of Smurf2 substrate selection, require further investigation.

Finally, no human samples were assessed in this study. Thus, there is no direct evidence that EP3 expression or the EP3-Smurf2-Smad7-Smad2/3 axis is similarly dysregulated in human cardiac fibrosis. The clinical relevance of DG041 remains to be established using human cardiac samples or human induced pluripotent stem (iPS)-derived cardiac cells and organoids.

## 6 Conclusions

The present study identifies CF-expressed EP3 as a critical mediator of sustained β-adrenergic activation-induced cardiac fibrosis and diastolic dysfunction. EP3 promotes Smurf2-mediated ubiquitination and degradation of Smad7, thereby weakening its inhibitory effect on TGF-β1-Smad2/3 signaling and facilitating CF activation and ECM production. Global and CF-specific *EP3* deletion, as well as preventive pharmacological blockade with DG041, attenuated ISO-induced fibrosis and diastolic dysfunction. These findings support further evaluation of EP3 antagonism as a strategy for improving cardiac fibrosis.

## Abbreviations

α-MyHC: α-Myosin heavy chain

α-SMA: α-smooth muscle actin

β-AR: β-adrenergic receptor

Akt: Protein kinase B

BV: Blood vessel

BW: Body weight

cAMP: Cyclic adenosine monophosphate

CF: Cardiac fibroblast

CHX: Cycloheximide

CM: Cardiomyocyte

CM-ISO: Conditioned medium

Co-IP: Co-Immunoprecipitation

CTGF: Connective tissue growth factor

cTnT: Cardiac troponin I

DAPI: 4’,6-diamidino-2-phenylindole

ECM: Extracellular matrix

E/A ratio: Early-to-atrial wave ratio

EF: Ejection fraction

eIF5: Eukaryotic translation initiation factor 5

EP: E-type prostanoid receptor

FS: Fractional shortening

H&E: Hematoxylin-eosin

HW: Heart weight

ISO: Isoproterenol

IS: Interstitium

IP: Immunoprecipitation

LV: Left ventricular

LVAW: Left ventricular anterior wall thickness

LVPW: Left ventricular posterior wall thickness

LVID: Left ventricular internal diameter

LV Vol: Left ventricular volume

PFA: Paraformaldehyde

PGE_2_: Prostaglandin E_2_

PI3K: Phosphatidylinositol 3-kinase

PKA: Protein kinase A

PM: Papillary muscle

PV: Panoramic view

SD: Sprague-Dawley

Smad: Small mother against decapentaplegic

Smurf2: Smad ubiquitination regulatory factor 2

S100A4: S100 calcium binding protein A4

TGF-β1: Transforming growth factor-β1

TL: Tibia length

Ub: Ubiquitin

VEGF: Vascular endothelial growth factor

WT: Wild-type

## Ethics approval and consent to participate

All animal care and experimental procedures were reviewed and approved by the Animal Care and Use Review Committee of East China Normal University (m+R20241108). The study conformed to the Guide for the Care and Use of Laboratory Animals published by the US National Institutes of Health.

## Funding

This work was supported by the National Key Research and Development Program of China (2022YFA1104500) and the National Natural Science Foundation of China (82270703, 82370423).

## Data Availability

All data generated or analyzed during this study are included in this published article and/or the supplementary information files.

## Footnotes

Appendix A Supplementary data associated with this article can be found in the online version at doi:10.1016/j.mmr.2026.100071.
