# Supplementary material for: Blockade of EP3 attenuates cardiac fibrosis by suppressing TGF-β1-Smad2/3 signaling through the Smurf2-mediated Smad7 ubiquitination
Source: Mil Med Res. 2026 Oct 6;13(1):100071. doi: 10.1016/j.mmr.2026.100071 (PMC13676097; doi:10.1016/j.mmr.2026.100071)
Supplement: Supplementary file 1 [file mmc1.pdf]

Methods

Cyclic adenosine monophosphate measurement

The levels of cyclic adenosine monophosphate (cAMP) in the cells were detected by cAMP ELISA kits (Jiangsu Meimian Industrial Co., Ltd., China). Cells were seeded in 6-well plates and pretreated with the protein kinase B (Akt) inhibitor MK2206 or protein kinase A (PKA) inhibitor H89 30 min before administration of the E-type prostanoid receptor 3 (EP3) antagonists. Cell lysates were subsequently collected and subjected to cAMP ELISA assay according to the manufacturer's instructions. Protein concentrations in the cell lysates were determined by the BCA assay according to the manufacturer's instructions.

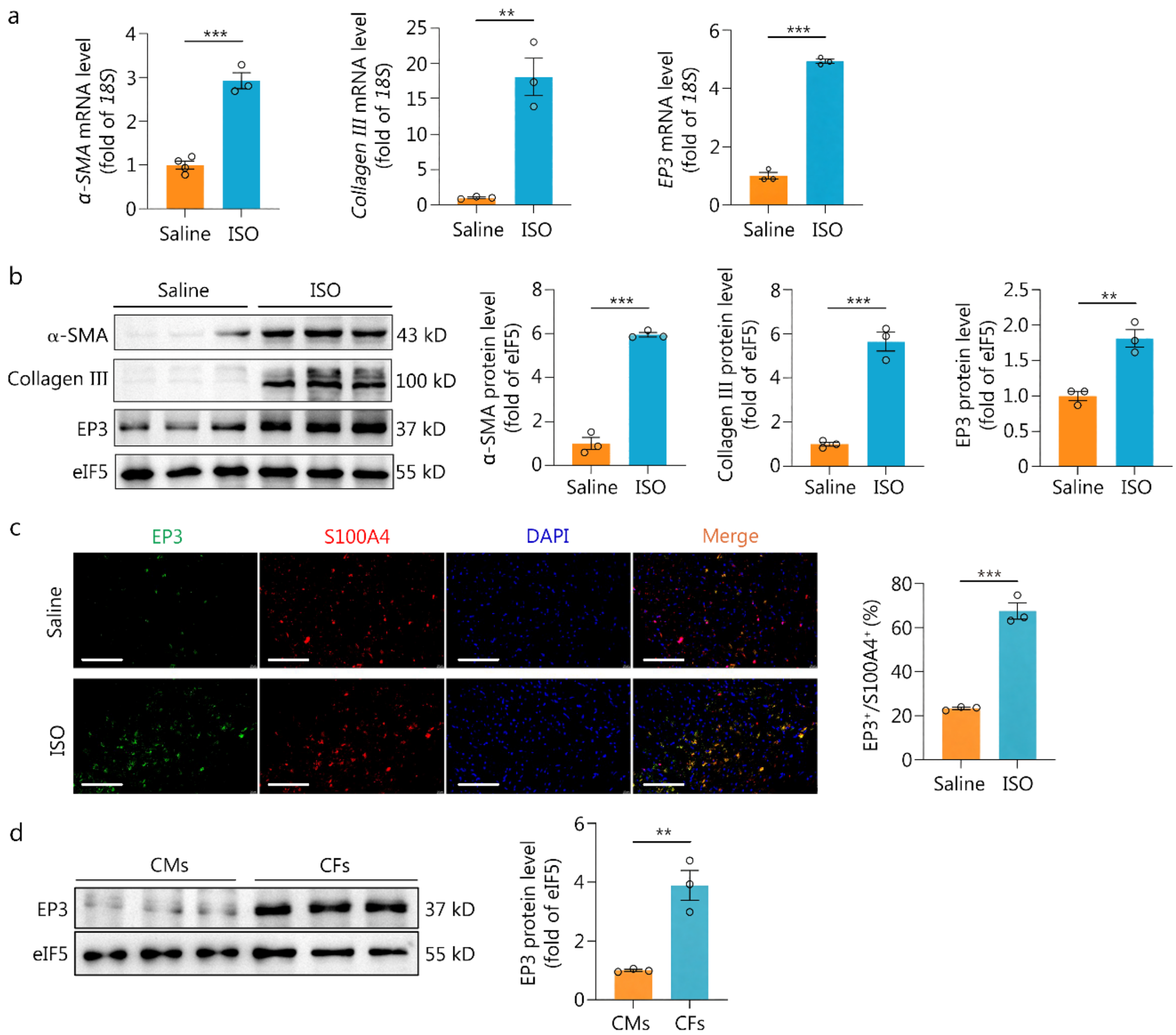

Fig. S1 Administration of isoproterenol (ISO) increases cardiac fibroblast EP3 expression in the CFs. **a** Ventricular tissues were subsequently collected for the analysis of mRNA levels of *α-SMA*, *collagen III*, and *EP3* in mouse ventricles after saline or ISO treatment for 7 d. *n*=3. **b** Western blotting was performed to detect the protein expression of α-SMA, collagen III, and EP3 in mouse ventricles after saline or ISO treatment for 7 d. eIF5 was used as an internal control. *n*=3. **c** Immunofluorescence staining showing the expression and the location of EP3 (in green) in mouse ventricles after saline or ISO treatment for 7 d. S100A4 was used to stain the cardiac fibroblasts (in red); DAPI stained the nuclei (in blue). The expression level of EP3 was analyzed relative to that of S100A4. Scale bar=100 μm. *n*=3. **d** Western blotting detected the EP3 protein expression levels of neonatal rat CMs and CFs, with eIF5 as an internal control. *n*=3. Data were presented as mean±SEM, $^{**}P<0.01$, $^{***}P<0.001$. SEM. Standard error of the mean; α-SMA. α-smooth muscle actin; 18S. 18S ribosomal RNA; EP3. E-type prostaglandin receptor 3; eIF5. Eukaryotic

translation initiation factor 5; S100A4. S100 calcium binding protein A4; DAPI. 4’,6-diamidino-2-phenylindole; CMs. Cardiomyocytes; CFs. Cardiac fibroblasts

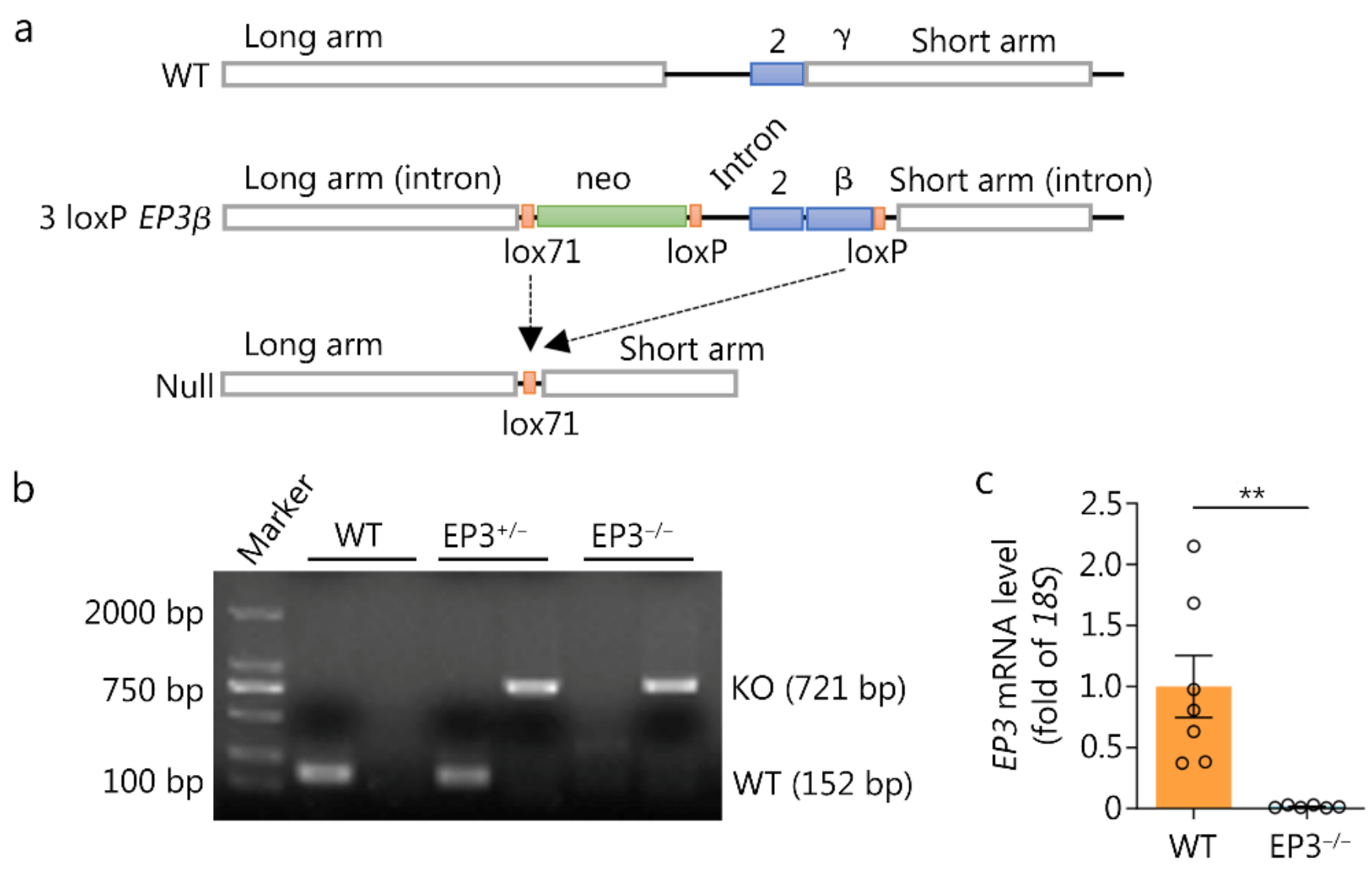

Fig. S2 Genotyping of the *EP3* gene knockout ($EP3^{-/-}$) mice. **a** The WT allele was altered by the correctly targeted 3 loxP *EP3β* knock-in vector, leading to the *EP3* null allele after *Cre* deletion at the outermost loxP site. **b** Genotyping of WT, *EP3* heterozygous ($EP3^{+/-}$), and *EP3* homozygous ($EP3^{-/-}$) mice. The PCR products of the WT allele (152 bp) and the knockout (KO) allele (721 bp) were separated using agarose gel electrophoresis. **c** The mRNA levels of the *EP3* in WT and $EP3^{-/-}$ mouse hearts. n=6−7. Data were presented as mean±SEM, $^{**}P<0.01$. SEM. Standard error of the mean; WT. Wild-type; EP3. E-type prostaglandin receptor 3; 18S. 18S ribosomal RNA

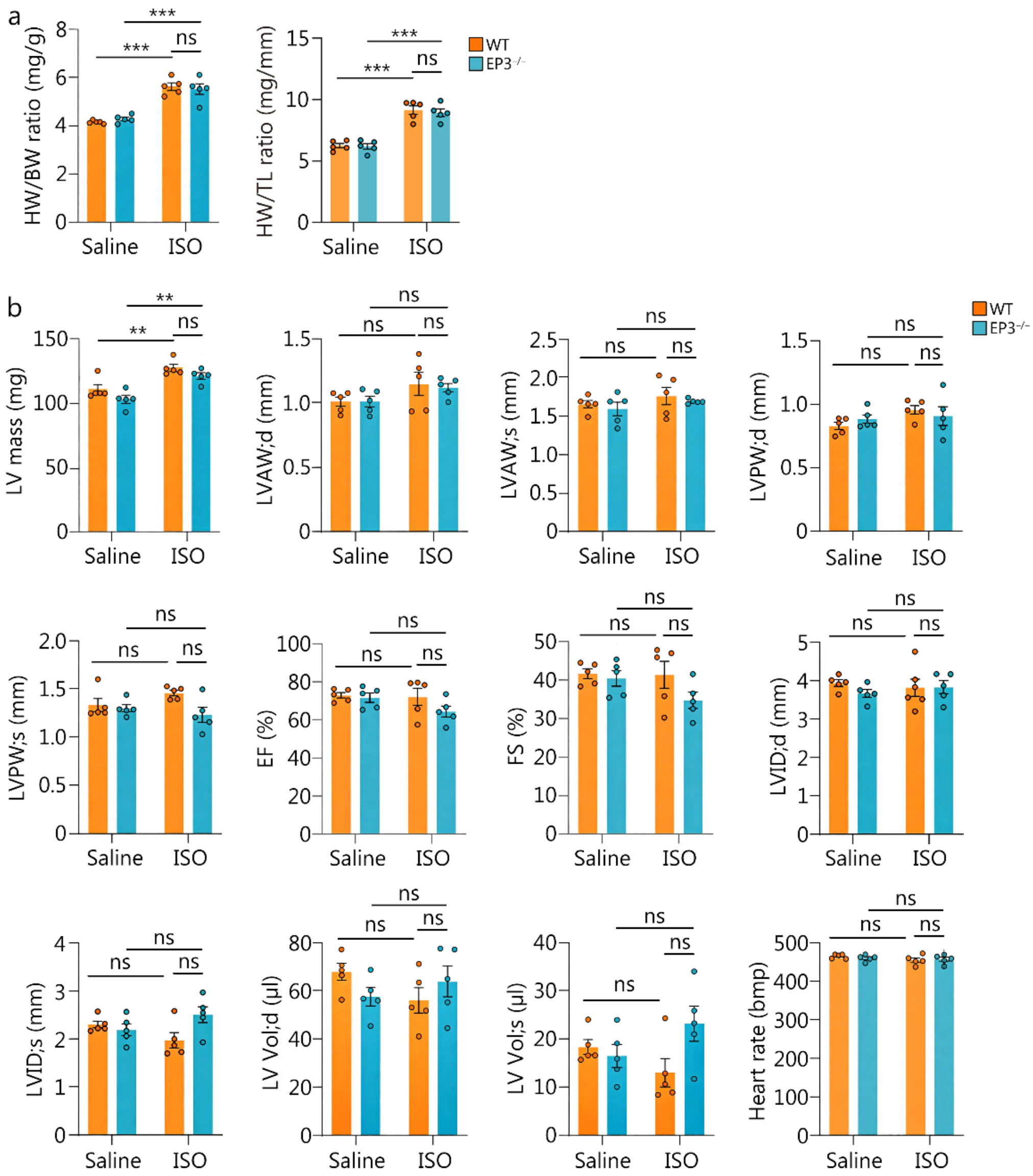

Fig. S3 Deletion of *EP3* has no effect on cardiac hypertrophy and systolic function in mice. **a** The heart weight (HW, mg) to body weight (BW, g) ratio (HW/BW ratio) and the HW (mg) to tibia length (TL, mm) ratio (HW/TL ratio) in WT and EP3$^{-/-}$ mice after saline or ISO injection for 7 d. *n*=5. **b** The echocardiography measurements of left ventricular mass (LV mass), left ventricular end-diastolic anterior wall thickness (LVAW;d), left ventricular end systolic anterior wall thickness (LVAW;s), left ventricular end-diastolic posterior wall thickness (LVPW;d), and left ventricular end-systolic posterior wall thickness (LVPW;s), ejection fraction (EF), fractional shortening (FS), left ventricular end-diastolic internal diameter (LVID;d) and left ventricular end-systolic internal diameter (LVID;s), left ventricular end-diastolic volume (LV Vol;d), and left ventricular end-systolic internal diameter (LV Vol;s) and heart rate in WT and EP3$^{-/-}$ mice after saline or ISO injection for

7 d. n=5. Data were presented as mean±SEM, $^{**}P<0.01$, $^{***}P<0.001$, ns non-significant. SEM. Standard error of the mean; EP3. E-type prostaglandin receptor 3; ISO. Isoproterenol

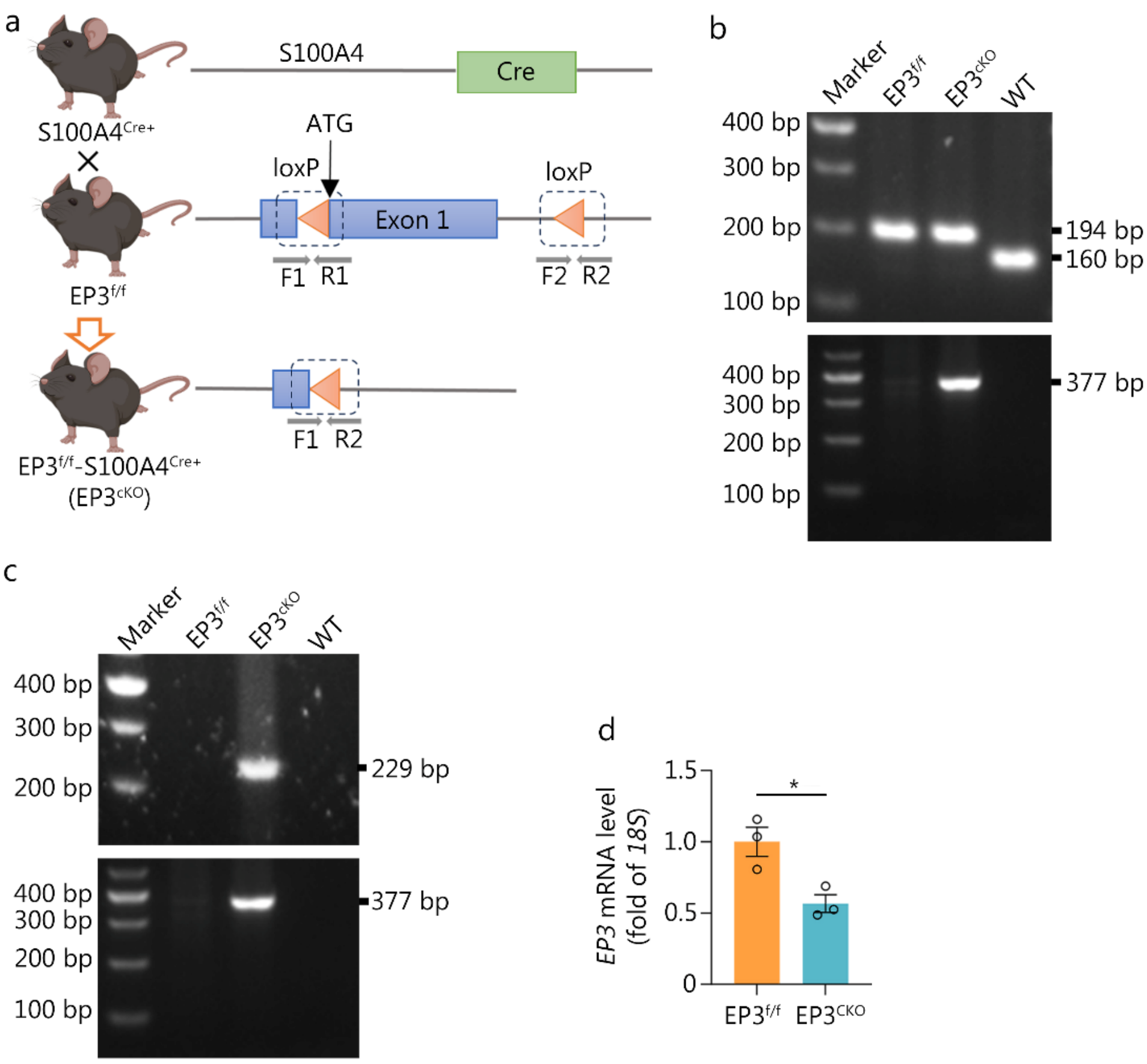

Fig. S4 Generation of the CF-*EP3*-specific knockout (EP3^{cKO}) mice. **a** Schematic showing the generation of the EP3^{cKO} mice. The floxed *EP3* allele (EP3$^{f/f}$) was modified by S100A4^{Cre+} at two loxP sites flanking exon 1 of the *EP3* allele. The F1/R1 and F2/R2 are the primers were designed to detect the loxP sites. F1 and R2 are the primers designed to detect the ablation of exon 1. **b** Genotyping of the EP3$^{f/f}$ and S100A4^{Cre+} using mouse tail DNA. The upper panel shows the floxed *EP3* allele (194 bp) and wild-type (WT) allele (160 bp), and the lower panel shows the S100A4^{Cre+} (377 bp). **c** PCR validation of the recombined floxed *EP3* allele (upper panel, 229 bp) and S100A4^{Cre+} (lower panel, 377 bp) using mouse heart DNA. **d** The mRNA levels of *EP3* in the EP3$^{f/f}$ and EP3^{cKO} mouse hearts. n=3. Data were presented as mean±SEM, $^{*}P<0.05$. SEM. Standard error of the mean; S100A4. S100 calcium binding protein A4; CF. Cardiac fibroblast; EP3. E-type prostaglandin receptor 3

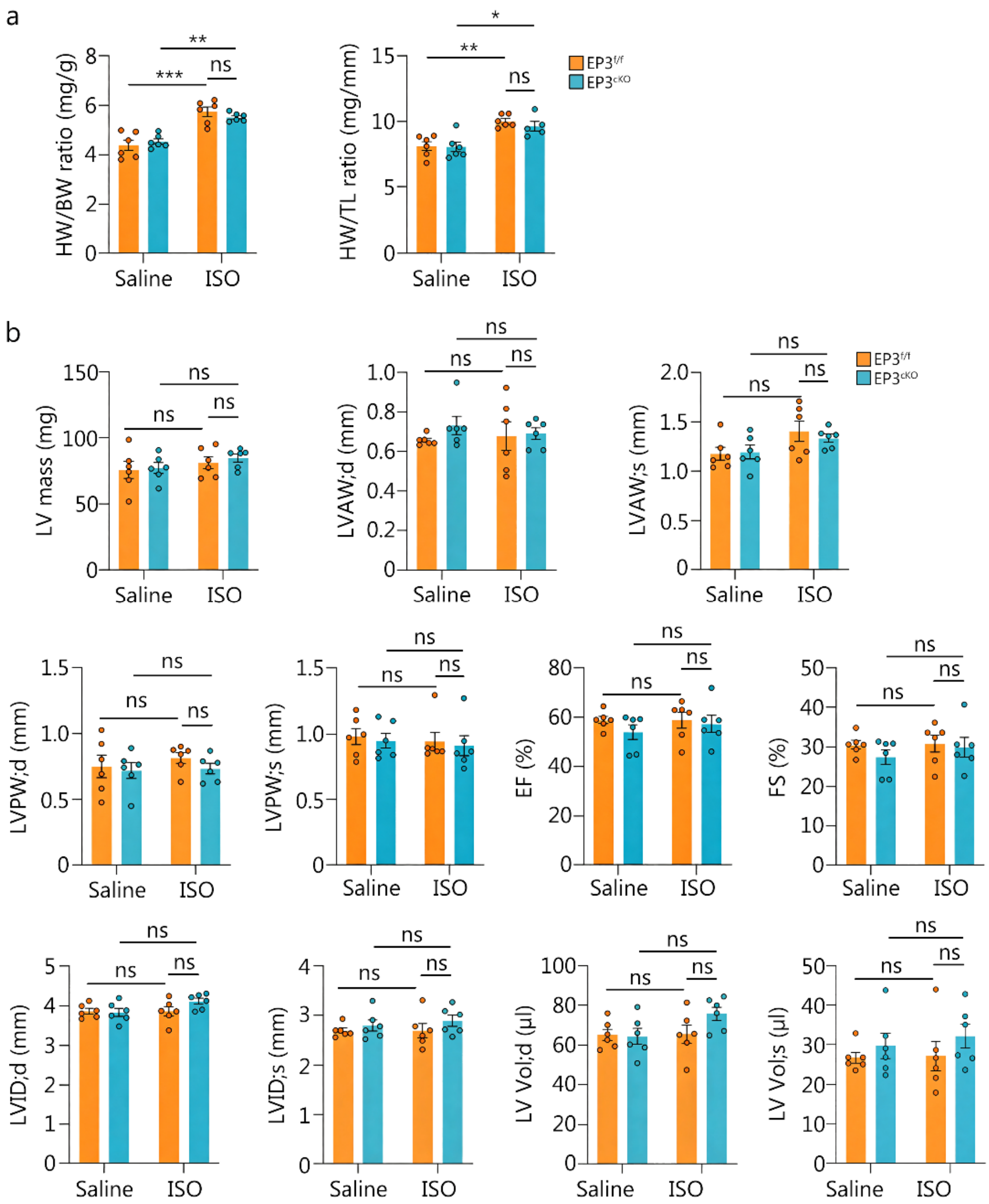

Fig. S5 Deletion of the CF-*EP3* has no effect on cardiac hypertrophy and systolic function in mice. **a** The heart weight (HW, mg) to body weight (BW, g) ratio (HW/BW ratio) and the HW (mg) to tibia length (TL, mm) ratio (HW/TL ratio) in the $EP3^{f/f}$ and $EP3^{cKO}$ mice after saline or ISO injection for 7 d. *n*=5−6. **b** The echocardiography measurements of left ventricular mass (LV mass), left ventricular end-diastolic anterior wall thickness (LVAW;d), left ventricular end systolic anterior wall thickness (LVAW;s), left ventricular end-diastolic posterior wall thickness (LVPW;d), left ventricular end-systolic posterior wall thickness (LVPW;s), ejection fraction (EF), fractional shortening (FS), left ventricular end-diastolic internal diameter (LVID;d), left ventricular end- systolic internal diameter (LVID;s), left ventricular end-diastolic volume (LV Vol;d), and left ventricular end-systolic internal diameter (LV Vol;s) in the $EP3^{f/f}$ and $EP3^{cKO}$ mice after saline or ISO

injection for 7 d. n=6. Data were presented as mean±SEM, $^{*}P<0.05$, $^{**}P<0.01$, $^{***}P<0.001$, ns non-significant. SEM. Standard error of the mean; CF. Cardiac fibroblast; ISO. Isoproterenol

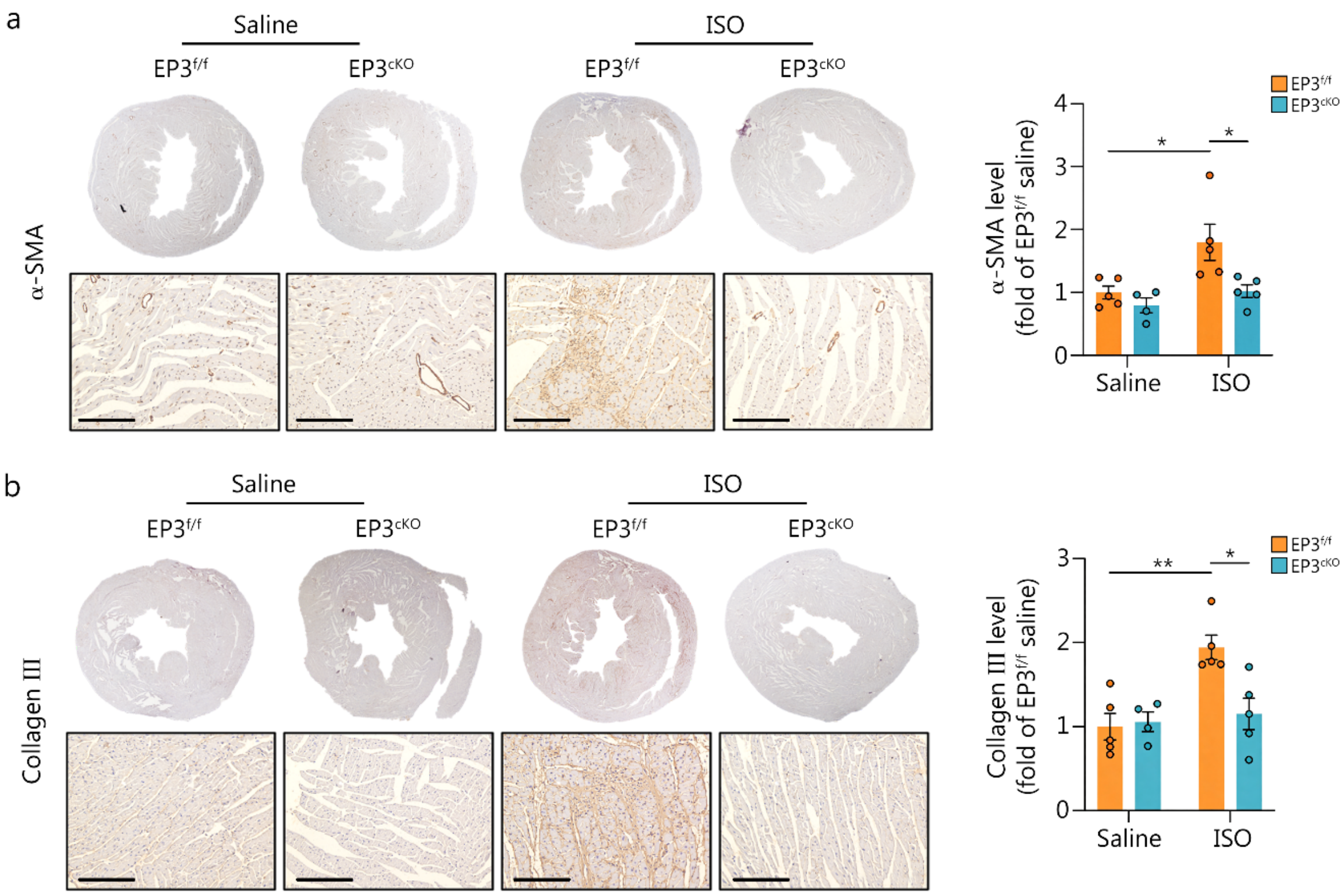

Fig. S6 Deletion of CF-*EP3* attenuates the ISO-induced increases in α-SMA and collagen III. Immunohistochemical staining of α-SMA and collagen III in the ventricular sections of the $EP3^{f/f}$ and $EP3^{cKO}$ mice treated with saline or ISO for 7 d. Scale bar=100 μm. *n*=4−5. Data were presented as mean±SEM, $^{*}P<0.05$, $^{**}P<0.01$. SEM. Standard error of the mean; EP3. E-type prostaglandin receptor 3; ISO. Isoproterenol; α-SMA. α-smooth muscle actin; CF. Cardiac fibroblast

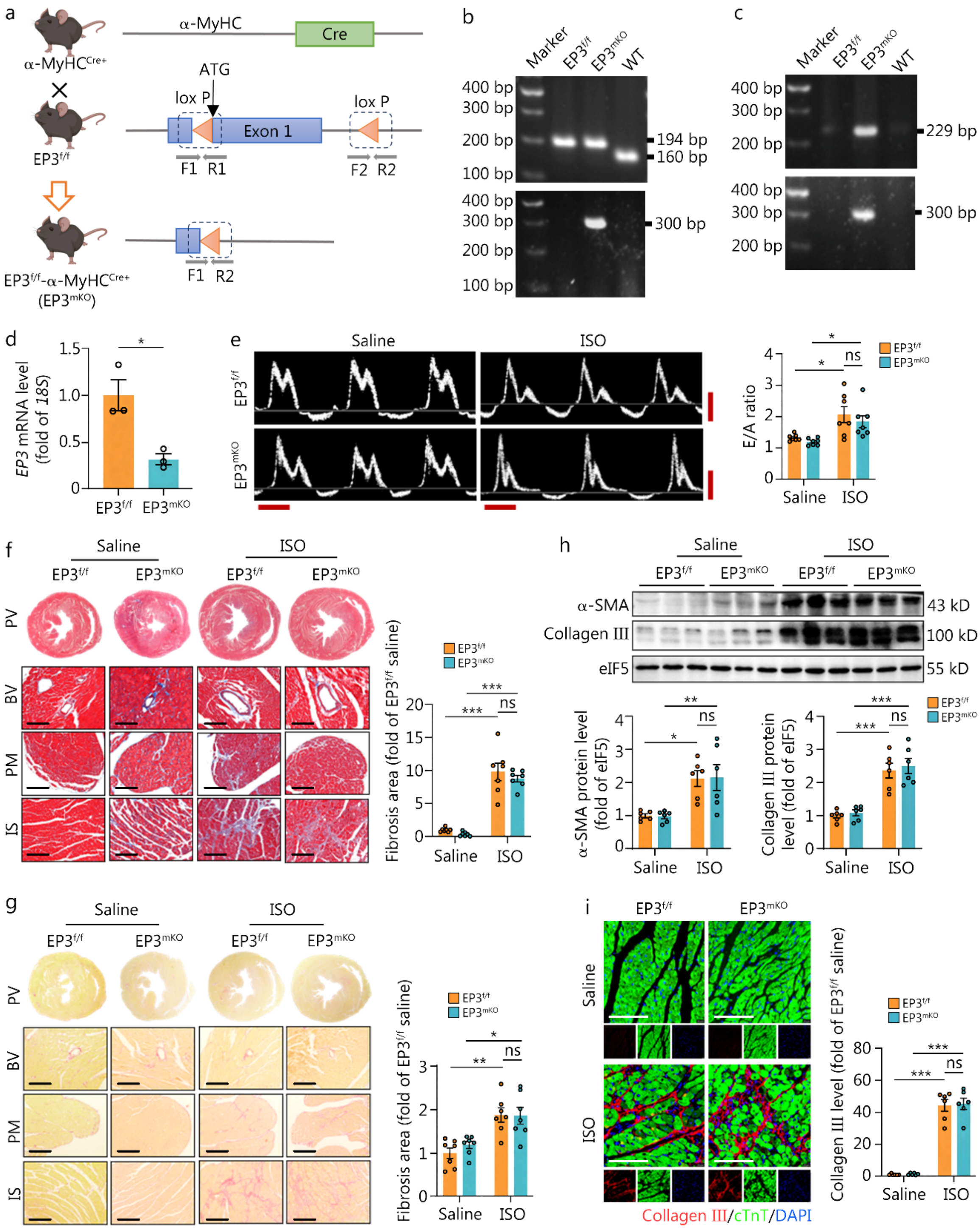

Fig. S7 Cardiomyocyte (CM)-specific *EP3* gene knockout mice (EP3^{mKO}) have no effect on ISO-induced cardiac diastolic dysfunction and fibrosis. **a** Schematic showing the generation of the EP3^{mKO} mice. The floxed *EP3* allele (EP3$^{f/f}$) was modified by α-MyHC^{Cre+} at two loxP sites flanking exon 1 of the *EP3* allele. The F1/R1 and F2/R2 primers were designed to detect the loxP sites. F1 and R2 are primers designed to detect the ablation

of exon 1. **b** Genotyping of the EP3$^{f/f}$ and α-MyHC^{Cre+} using mouse tail DNA. The upper panel shows the floxed *EP3* allele (194 bp) and WT allele (160 bp), the lower panel shows the α-MyHC^{Cre+} (300 bp). **c** PCR validation of the recombined floxed EP3 allele (upper panel, 229 bp) and α-MyHC^{Cre+} (lower panel, 300 bp) using mouse heart DNA. **d** The mRNA levels of *EP3* in the EP3$^{f/f}$ and EP3^{mKO} mouse hearts were examined by quantitative real-time PCR. *n*=3. **e** The Doppler color ultrasound measurements of the peak velocity flow in early diastole (E, m/s) and late diastole by atrial contraction (A, m/s) ratio (E/A ratio) in EP3$^{f/f}$ and EP3^{mKO} mice injected with saline or ISO for 7 d. The horizontal scale bar represents a time course of 100 ms. The vertical scale represents a blood flow velocity of 4 m/s. *n*=7. Masson's trichrome staining (**f**) and Sirius Red staining (**g**) of the ventricular sections of the EP3$^{f/f}$ and EP3^{mKO} mice treated with saline or ISO for 7 d. Scale bar=100 μm. *n*=7. **h** Western blotting analysis of α-SMA and collagen III protein levels in the ventricles of the EP3$^{f/f}$ and EP3^{mKO} mice treated with saline or ISO for 7 d, with eIF5 as internal control. *n*=6. **i** Immunofluorescence staining of collagen III (in red) and cTnT (in green) in the ventricular sections of the EP3$^{f/f}$ and EP3^{mKO} mice treated with saline or ISO for 7 d. DAPI stained the nuclei (in blue). Scale bar=100 μm. *n*=6. Data were presented as mean ± SEM, $^{*}P<0.05$, $^{**}P<0.01$, $^{***}P<0.001$, ns non-significant. SEM. Standard error of the mean; α-MyHC. α-Myosin heavy chain; EP3. E-type prostaglandin receptor 3; WT. Wild-type; ISO. Isoproterenol; α-SMA. α-smooth muscle actin; eIF5. Eukaryotic translation initiation factor 5; cTnT. Cardiac troponin I; DAPI. 4',6-diamidino-2-phenylindole. PV. Panoramic view; BV. Blood vessels; PM. Papillary muscle; IS. Interstitium

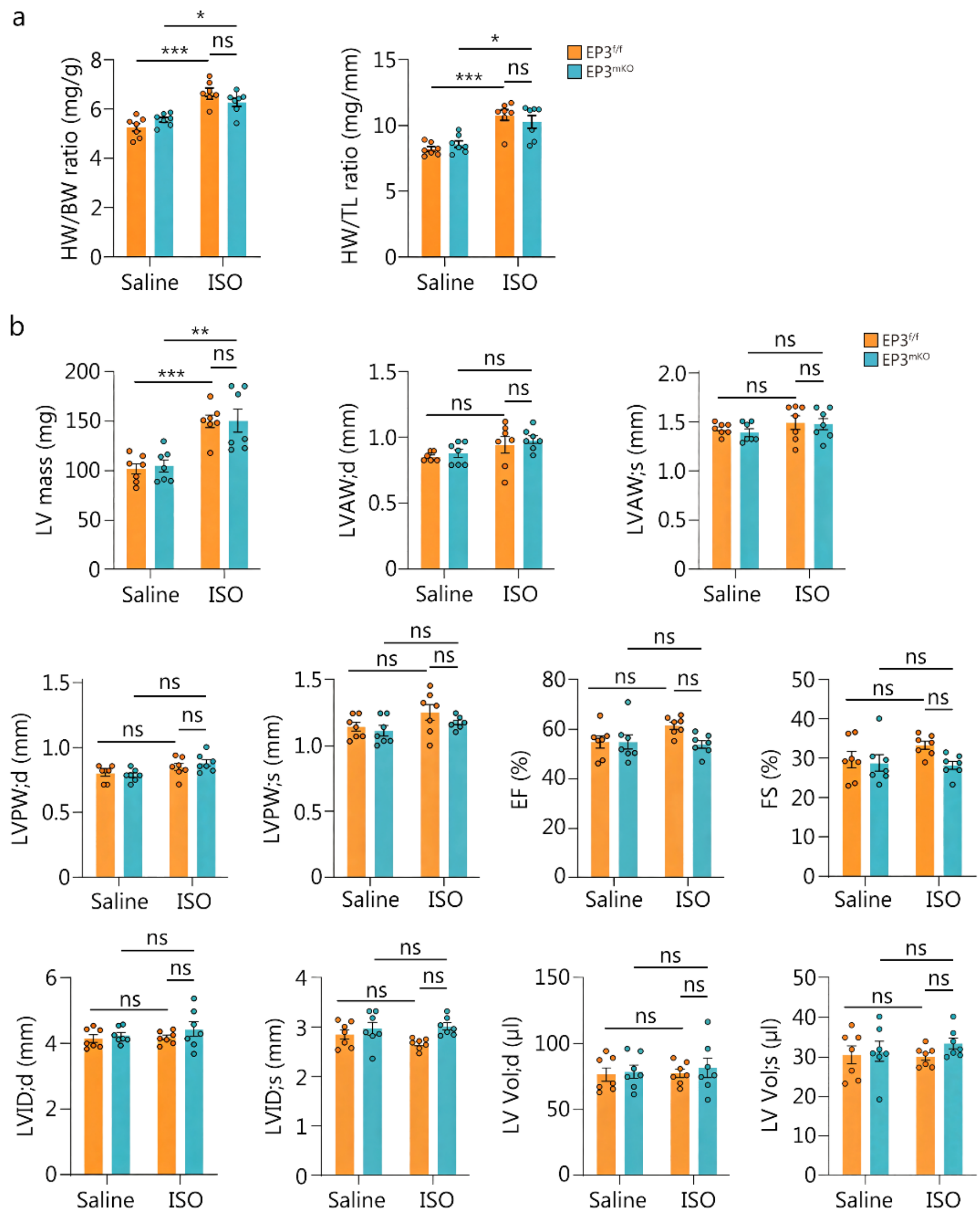

Fig. S8 Deletion of the CM-*EP3* has no effect on cardiac hypertrophy and systolic function in mice. **a** The heart weight (HW, mg) to body weight (BW, g) ratio (HW/BW ratio) and the HW (mg) to tibia length (TL, mm) ratio (HW/TL ratio) in the $EP3^{f/f}$ and $EP3^{mKO}$ mice after saline or ISO injection for 7 d. *n*=7. **b**. Echocardiography measurements of left ventricular mass (LV mass), left ventricular end-diastolic anterior wall thickness (LVAW;d), left ventricular end systolic anterior wall thickness (LVAW;s), left ventricular end-diastolic posterior wall thickness (LVPW;d), left ventricular end-systolic posterior wall thickness (LVPW;s), ejection fraction (EF), fractional shortening (FS), left ventricular end-diastolic internal diameter (LVID;d), left ventricular end- systolic internal diameter (LVID;s), left ventricular end-diastolic volume (LV Vol;d), and left ventricular end-

systolic internal diameter (LV Vol;s) in the $EP3^{f/f}$ and $EP3^{mKO}$ mice after saline or ISO injection for 7 d. *n*=7. Data were presented as mean±SEM, $^{*}P<0.05$, $^{**}P<0.01$, $^{***}P<0.001$. SEM. Standard error of the mean; CM. Cardiomyocyte; EP3. E-type prostaglandin receptor 3; ISO. Isoproterenol

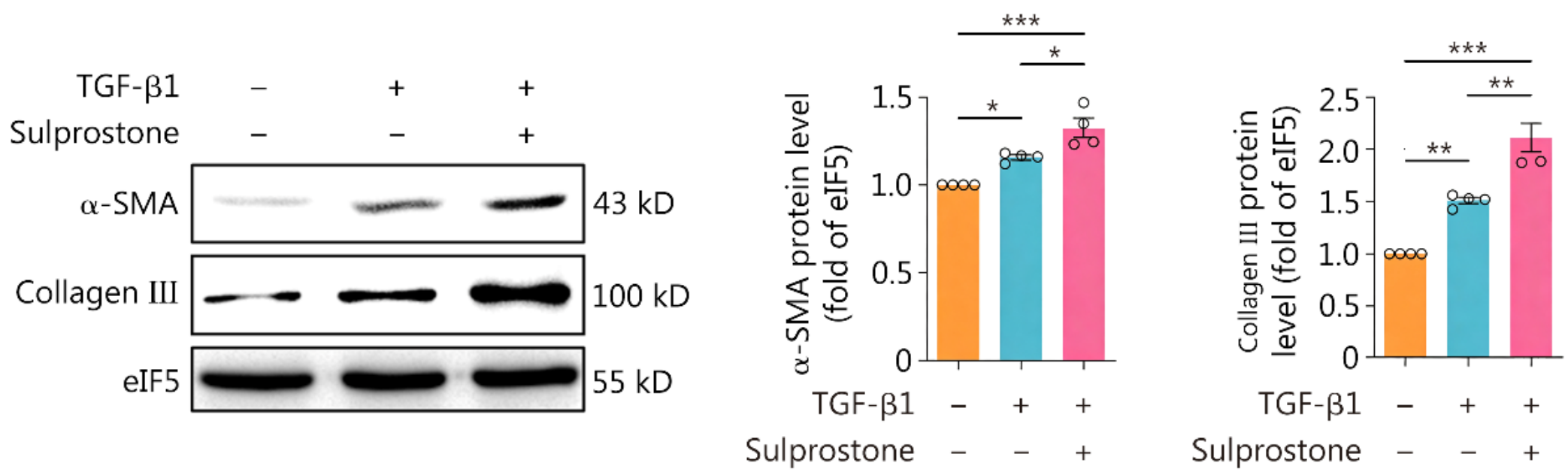

Fig. S9 Sulprostone treatment enhances TGF-β1-mediated CF activation and ECM production. The protein expression of α-SMA and collagen III after sulprostone (1 μmol/L) treatment for 30 min, followed by treatment with TGF-β1 (10 ng/ml) for another 24 h in the CFs. eIF5 was used as an internal control. Quantitative analysis was performed using ImageJ software. *n*=4. Data were presented as mean±SEM, $^{*}P<0.05$, $^{**}P<0.01$, $^{***}P<0.001$. SEM. Standard error of the mean; TGF-β1. Transforming growth factor-β1; α-SMA. α-smooth muscle actin; eIF5. Eukaryotic translation initiation factor 5; CF. Cardiac fibroblast

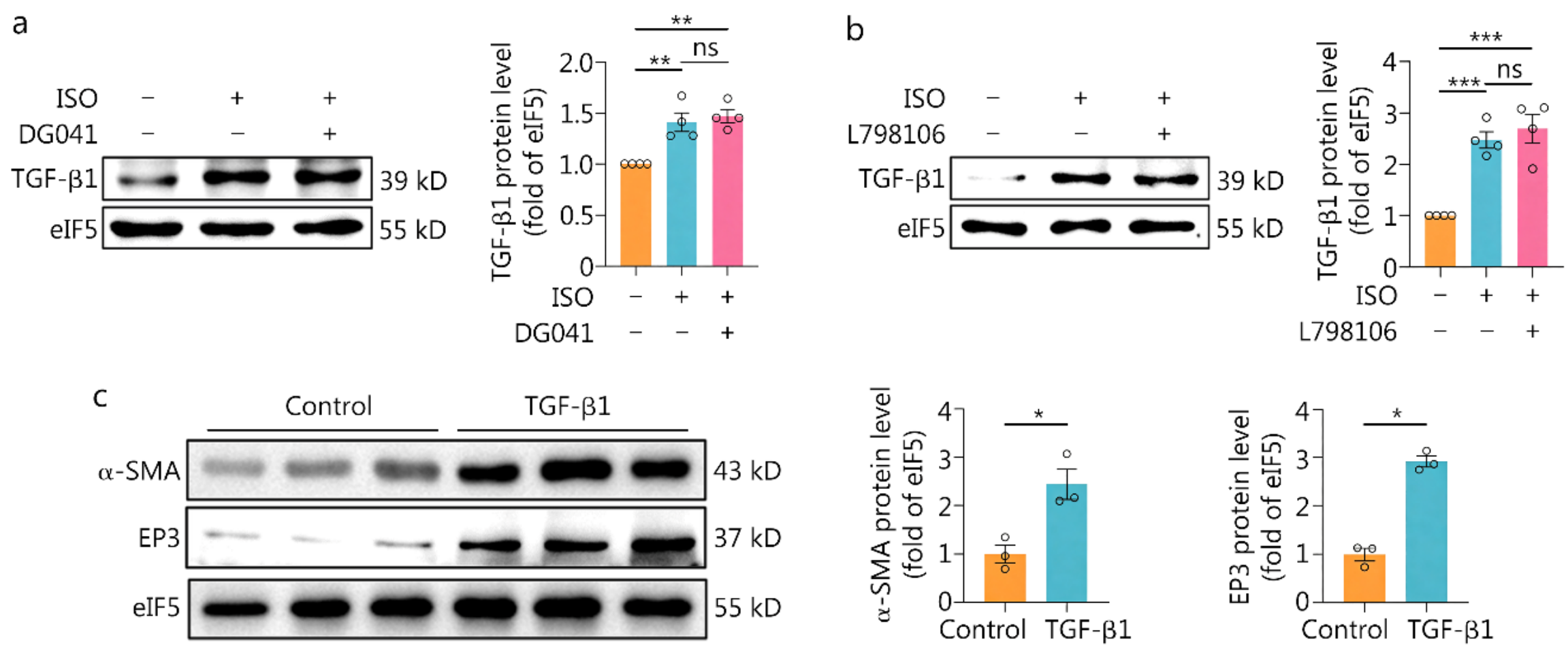

Fig. S10 Blockade of EP3 fails to suppress ISO-induced increase of TGF-β1 in the CMs, and TGF-β1 upregulates EP3 expression in the CFs. **a** The protein expression of TGF-β1 after pretreatment of DG041 (1 μmol/L) for 30 min and then treated with ISO (20 μmol/L) for another 24 h in the CMs. eIF5 was used as an internal control. *n*=4. **b** The protein expression of TGF-β1 after pretreatment with L798106 (1 μmol/L) for 30 min and then treated with ISO (20 μmol/L) for another 24 h in the CMs. eIF5 was used as an internal control. *n*=4. **c** The protein expression of α-SMA and EP3 after treatment with TGF-β1 (10 ng/ml) for 24 h in the CFs, with eIF5 as an internal control. *n*=3. Data were presented as mean±SEM, $^{*}P<0.05$, $^{**}P<0.01$, $^{***}P<0.001$, ns non-significant. SEM. Standard error of the mean; ISO. Isoproterenol; TGF-β1. Transforming growth factor-β1; α-SMA. α-smooth muscle actin; EP3. E-type prostaglandin receptor 3; eIF5. Eukaryotic translation initiation factor 5; CFs. Cardiac fibroblasts; CMs. Cardiomyocytes

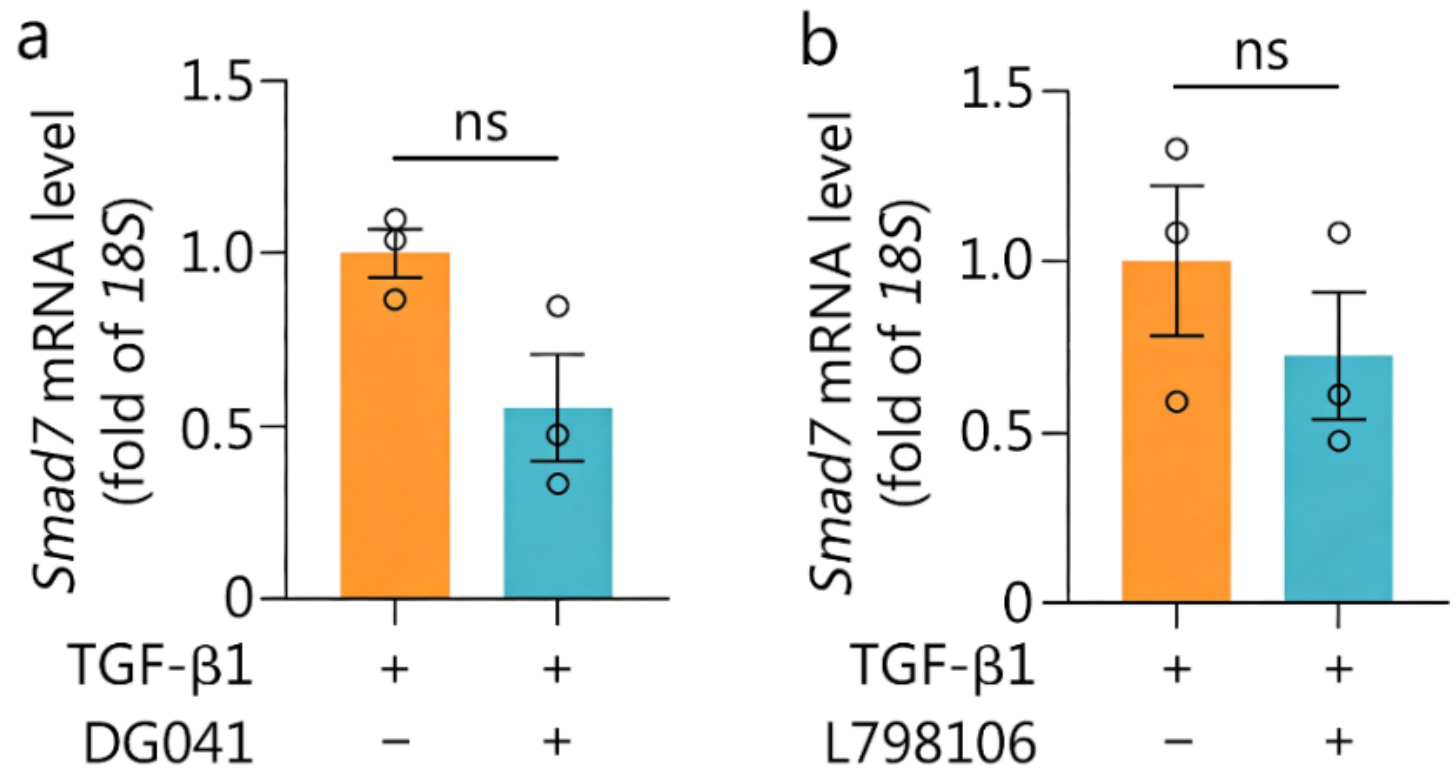

Fig. S11 The inhibition of EP3 has no significant effect on *Smad7* mRNA levels in the CFs. The mRNA expression of *Smad7* after treatment with DG041 (1 μmol/L) or L798106 (1 μmol/L) plus TGF-β1 (10 ng/ml) for 3 h in CFs. *n*=3. Data were presented as mean±SEM. ns. Non-significant. SEM. Standard error of the mean; EP3. E-type prostaglandin receptor 3; Smad7. Small mother against decapentaplegic7; CFs. Cardiac fibroblasts; TGF-β1. Transforming growth factor-β1

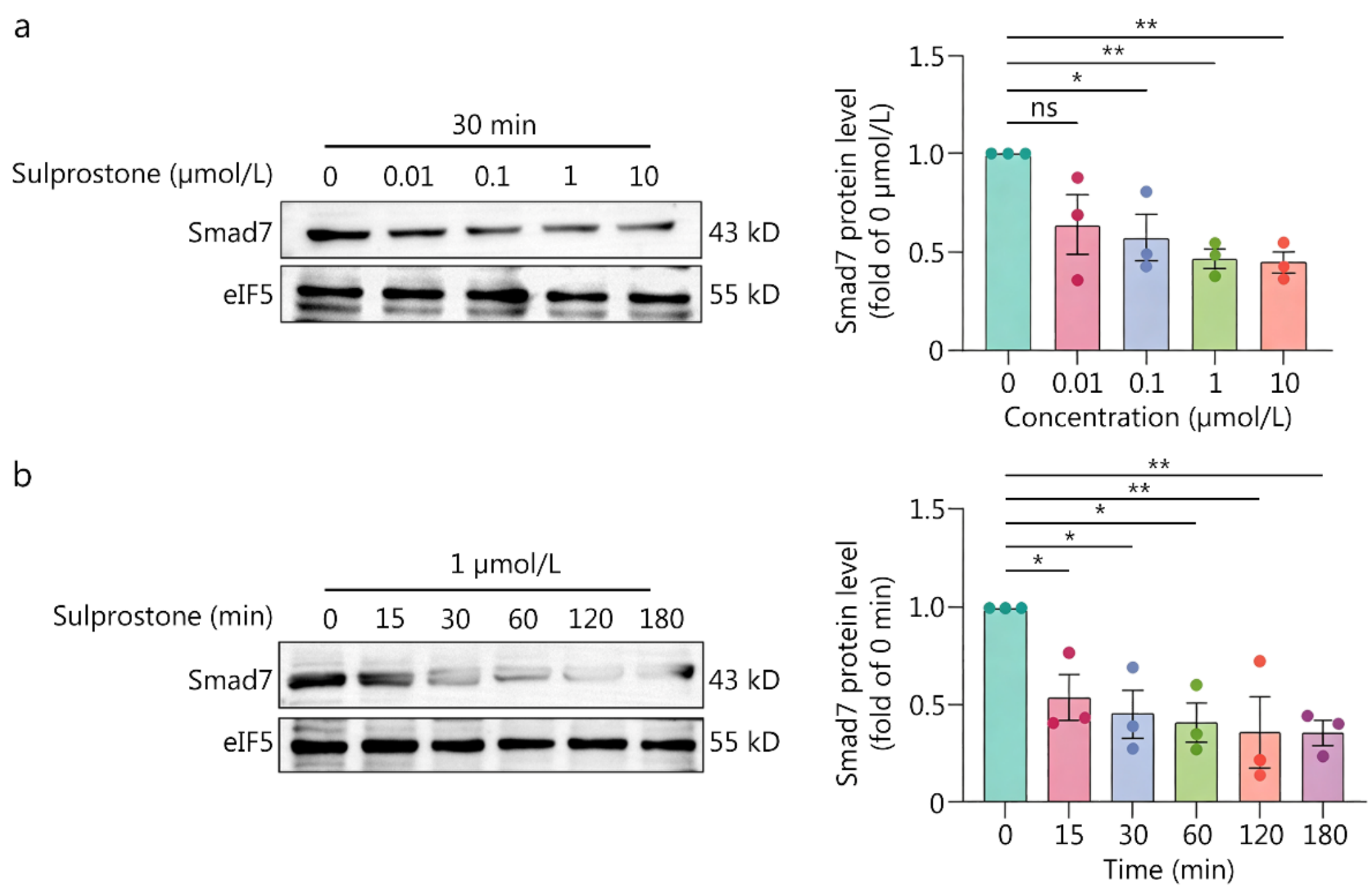

Fig. S12 Treatment with sulprostone decreases Smad7 expression in a concentration- and time-dependent manner in the CFs. **a** The CFs were treated with various concentrations of sulprostone (0, 0.01, 0.1, 1, 10 μmol/L) for 30 min. eIF5 was used as an internal control. **b** The CFs were treated with 1 μmol/L sulprostone for various time periods (0, 15, 30, 60, 120, 180 min). eIF5 was used as an internal control. n=3. Data were presented as mean±SEM, $^{*}P$<0.05, $^{**}P$<0.01, ns non-significant. SEM. Standard error of the mean; Smad7. small mother against decapentaplegic7; eIF5. Eukaryotic translation initiation factor 5; CFs. Cardiac fibroblasts

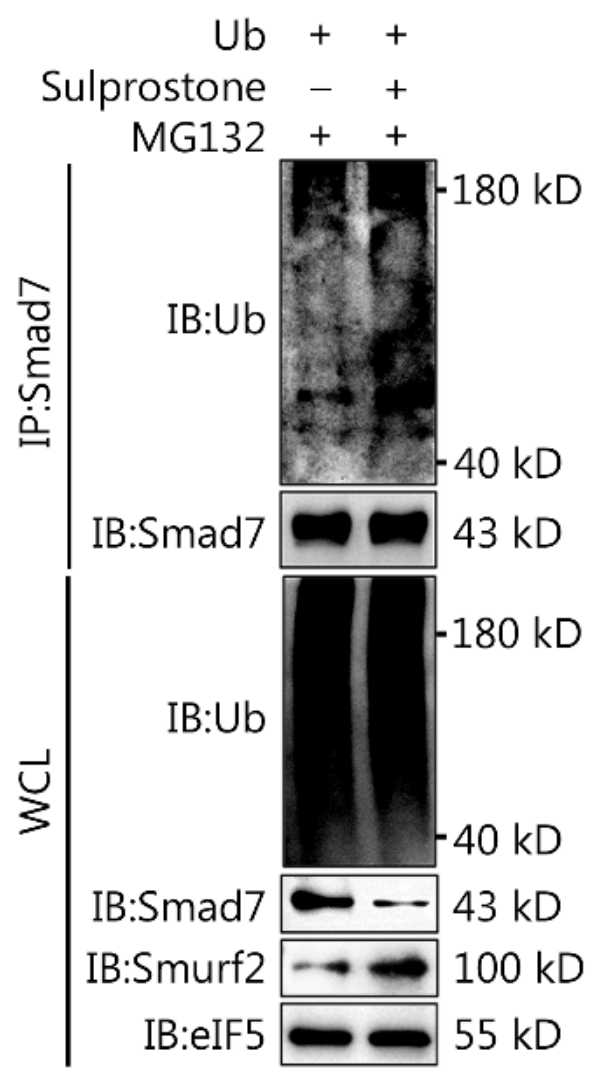

Fig. S13 Treatment with sulprostone increases Smad7 ubiquitination in the CFs. The CFs were transfected with a ubiquitin overexpressing vector for 36 h and then pretreated with sulprostone (1 μmol/L) for 30 min, followed by treatment with MG132 (1 μmol/L) for another 3 h. Co-immunoprecipitation (Co-IP) was performed using the rabbit anti-Smad7 primary antibody, and the immunoprecipitated proteins were blotted with an anti-ubiquitin primary antibody. The whole cell lysate (WCL) was used to examine the total protein levels of ubiquitin, Smad7, and Smurf2. eIF5 was used as an internal control. Ub. Ubiquitin; Smad7. Small mother against decapentaplegic7; Smurf2. Smad ubiquitination regulatory factor 2; eIF5. Eukaryotic translation initiation factor 5; CFs. Cardiac fibroblasts

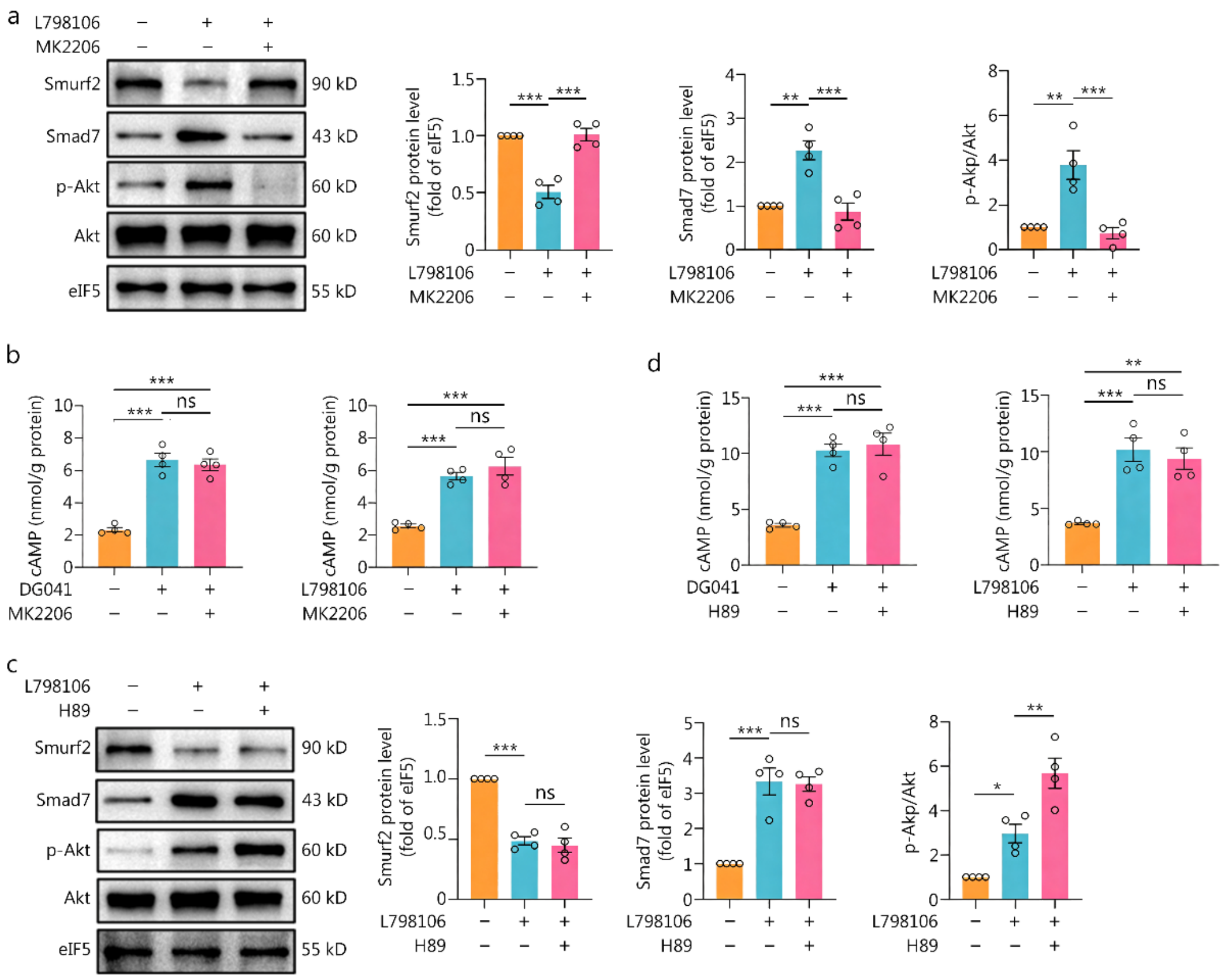

Fig. S14 Blockade of EP3 downregulates Smurf2 protein expression in an Akt-dependent manner. **a** The protein expression of Smurf2, Smad7, p-Akt, and Akt after pretreatment of MK2206 (5 μmol/L) for 30 min and then treated with L798106 (1 μmol/L) for 3 h in the CFs, with eIF5 as an internal control. *n*=3. **b** The intracellular cAMP levels after pretreatment with MK2206 (5 μmol/L) for 30 min and then treated with DG041 (1 μmol/L) or L798106 (1 μmol/L) for 3 h in the CFs, with calibration based on protein concentration. *n*=4 . **c** Western blotting assay was performed to examine the expression of Smurf2, Smad7, p-Akt, and Akt after pretreatment with H89 (10 μmol/L) for 30 min and then treated with L798106 (1 μmol/L) for 3 h in the CFs, with eIF5 as an internal control. *n*=4. **d** The intracellular cAMP levels after pretreatment with H89 (10 μmol/L) for 30 min and then treated with DG041 (1 μmol/L) or L798106 (1 μmol/L) for 3 h in the CFs, with calibration based on protein concentration. *n*=4. Data were presented as mean±SEM, $^{**}P<0.01$, $^{***}P<0.001$, ns non-significant. SEM. Standard error of the mean; Smurf2. Smad ubiquitination regulatory factor 2; Smad7. Small mother against decapentaplegic7; Akt. Protein kinase B; eIF5. Eukaryotic translation initiation factor 5; cAMP. Cyclic AMP; CFs. Cardiac fibroblasts

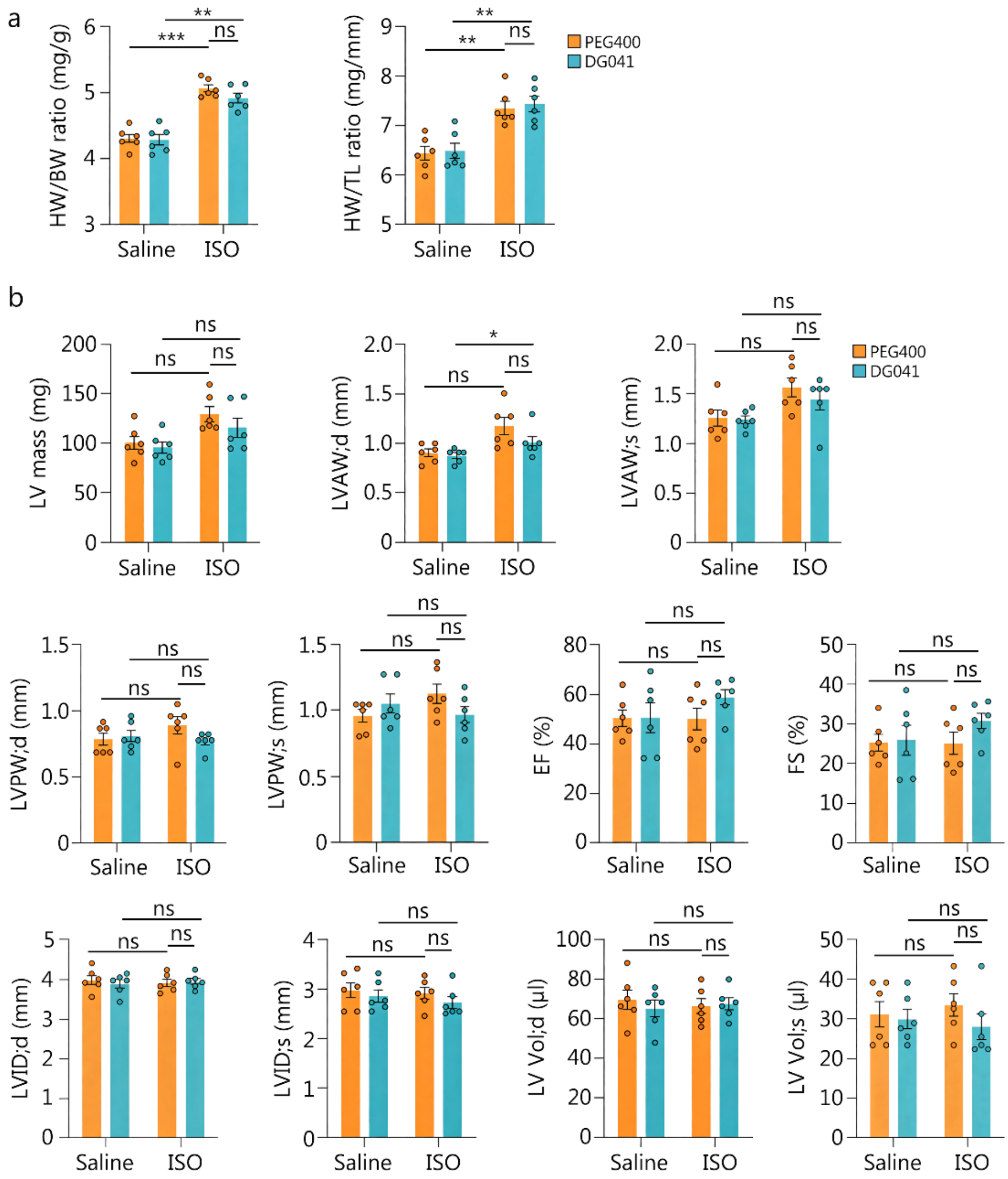

Fig. S15 Pharmacological blockade of EP3 has no effect on cardiac hypertrophy and systolic function in mice. **a** The heart weight (HW, mg) to body weight (BW, g) ratio (HW/BW ratio) and the HW (mg) to tibia length (TL, mm) ratio (HW/TL ratio) in PEG400- and DG041-treated mice after saline or ISO injection for 7 d. *n*=6. **b** Echocardiography measurements of left ventricular mass (LV mass), left ventricular end-diastolic anterior wall thickness (LVAW;d), left ventricular end systolic anterior wall thickness (LVAW;s), left ventricular end-diastolic posterior wall thickness (LVPW;d), left ventricular end-systolic posterior wall thickness (LVPW;s), ejection fraction (EF), fractional shortening (FS), left ventricular end-diastolic internal diameter (LVID;d), left ventricular end- systolic internal diameter (LVID;s), left ventricular end-diastolic volume (LV Vol;d), and left ventricular end-systolic internal diameter (LV Vol;s) in PEG400- and DG041-treated mice

after saline or ISO injection for 7 d. n=6. Data were presented as mean±SEM, $^{*}P<0.05$, $^{**}P<0.01$, $^{***}P<0.001$, ns non-significant. SEM. Standard error of the mean; ISO. Isoproterenol; EP3. E-type prostaglandin receptor 3

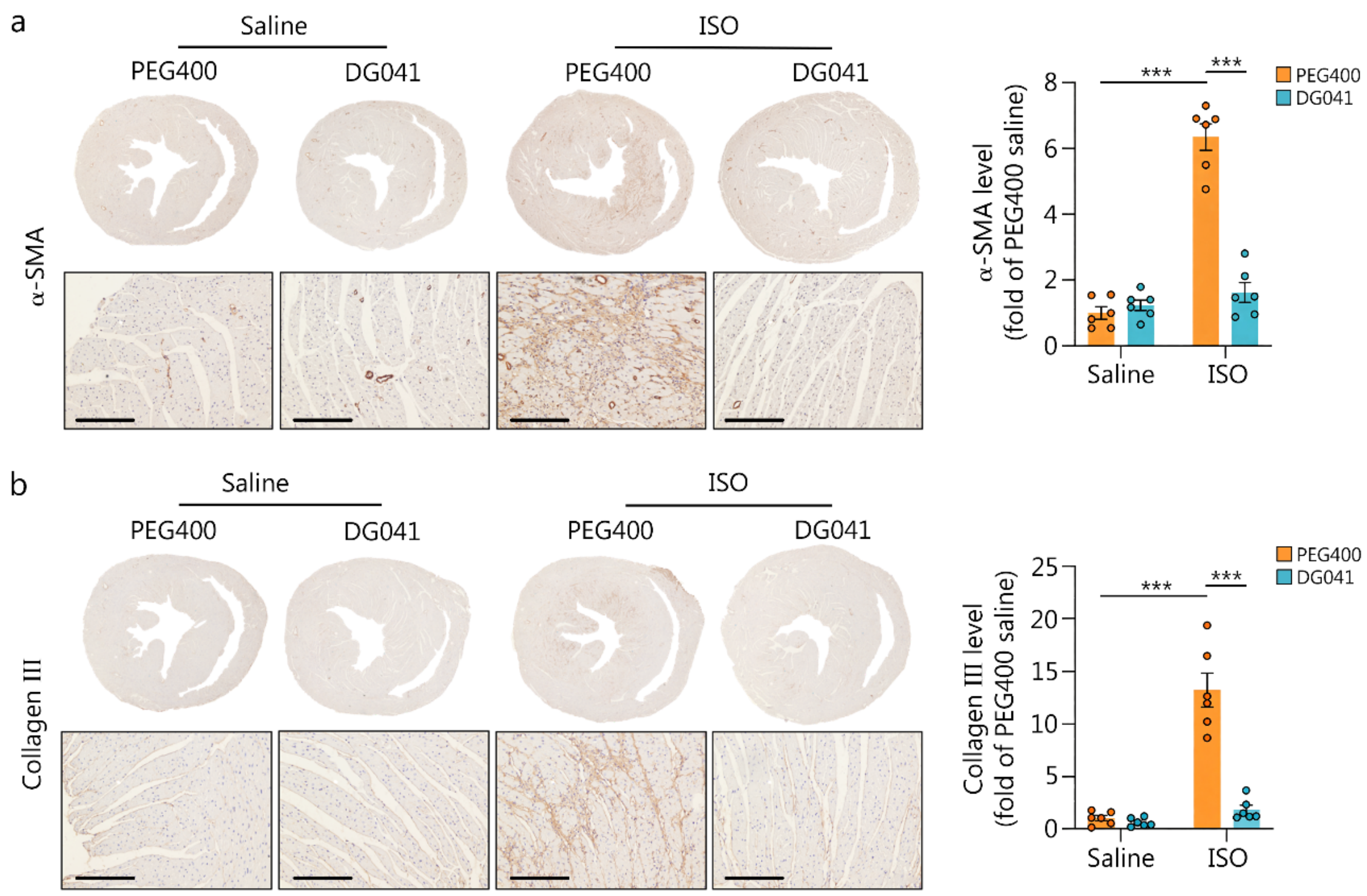

Fig. S16 Pharmacological blockade of EP3 suppresses ISO-induced cardiac α-SMA and collagen III expression *in vivo*. Immunohistochemical staining of α-SMA and collagen III in the ventricular sections of the PEG400- or DG041-treated mice injected with saline or ISO for 7 d. Scale bar=100 μm. *n*=6. Data were presented as mean±SEM, $^{***}P<0.001$. SEM. Standard error of the mean; ISO. Isoproterenol; α-SMA. α-smooth muscle actin; EP3. E-type prostaglandin receptor 3

Table S1 The primers used for genotyping

Gene	Sequence (5'-3')
EP3-WT-F	GCTGTCTCCAGTTGCTCTA
EP3-WT-R	TGCCTCAGTCCATAAGGGTTAGGG
EP3-KO-F	TGGCACAGAAAGGATTATCTA
EP3-KO-R	AACGCTTGTCAAATGTTCAT
EP3flox-F1	TGCACTCTGCTGCTATCCCGC
EP3flox-R1	CCACGGACACGGAGCCGCAG
EP3flox-F2	ATGATGACAACTCTAGCTCAACCT
EP3flox-R2	CAGAAAGACAGTTTGCCAAGAAGG
S100A4-Cre-F	TGCCACGACCAAGTGACAGCAATG
S100A4-Cre-R	ACCAGAGACGGAAATCCATCGCTC
αMyHC-Cre-F	ATGACAGACAGATCCCTCCTATCTCC
αMyHC-Cre-R	CTCATCACTCGTTGCATCATCGAC

EP3. E-type prostanoid receptor 3; WT. Wild-type; KO. Knockout; S100A4. S100 calcium binding protein A4; α-MyHC. α-Myosin heavy chain; F. Forward primer; R. Reverse primer

Table S2 The primers used for real-time PCR

**Gene**	**Sequence (5’-3’)**
Mouse-*α-SMA*-F	GTACCACCATGTACCCAGGC
Mouse-*α-SMA*-R	GCTGGAAGGTAGACAGCGAA
Mouse-*Collagen III*-F	TCAAGGCTGAAGGAAACAGCA
Mouse-*Collagen III*-R	GATGGGTAGTCTCATTGCC
Mouse-*EP3*-F	GGATCATGTGTGTGCTGTCC
Mouse-*EP3*-R	GCAGAACTTCCGAAGAAGGA
Mouse-18S-F	GAAACGGCTACCACATCCAAGG
Mouse-18S-R	GCCCTCCAATGGATCCTCGTTA
Rat-*Smad7*-F	GATCCGGCCACCTGAACACT
Rat-*Smad7*-R	GTGCCAAAGGTCACCACCATC
Rat-*18S*-F	TGCGGAAGGATCATTAACGGA
Rat-18S-R	AGTAGGAGAGGAGCGAGCGACC

α-SMA. α-smooth muscle actin; EP3. E-type prostanoid receptor 3; Smad7. Small mother against decapentaplegic 7; 18S. 18S ribosomal RNA; F. Forward primer; R. Reverse primer
